# Intergenerational association of early childhood education and interpersonal violence: a retrospective cohort study

**DOI:** 10.1186/s40621-026-00669-2

**Published:** 2026-03-13

**Authors:** Julia P. Schleimer, Anjum Hajat, Gail Joseph, Min Sun, Frederick Rivara, Ali Rowhani-Rahbar

**Affiliations:** 1 Department of Epidemiology, School of Public Health, University of Washington, Seattle, WA USA; 2 Center for Firearm Injury Prevention, Department of Pediatrics, School of Medicine, University of Washington, Seattle, WA USA; 3 College of Education, University of Washington, Seattle, WA USA

**Keywords:** Violence, Intergenerational, Early childhood education, Head Start

## Abstract

**Background:**

There is a need for research on the effects of primary prevention strategies that modify upstream social and economic drivers of interpersonal violence, especially intergenerationally. We examined the association between maternal exposure to Head Start (a high-quality preschool program for low-income children) and offsprings’ risk of violence, hypothesizing that benefits would be greatest for Black and Hispanic/Latino male offspring.

**Methods:**

This was a retrospective cohort study using National Longitudinal Survey of Youth 1979 (NLSY79) and intergenerationally linked Child and Young Adult Cohort (NLSCYA) data. Offspring were born between 1970 and 2014, with follow-up from 1988 to 2020. The exposure was Head Start availability in the birth county when mothers were aged 3–5 years. Outcomes were self-reported serious fighting (ages 10–17) and assault conviction (ages 15–25) among offspring. We excluded NLSCYA respondents whose mother was born outside the United States/moved to the United States after aged 5, who were never age eligible to answer the questions about violence, and whose maternal grandmother had a high school degree or higher (i.e., limiting the sample to NLSCYA respondents whose mother was most likely to have attended Head Start).

**Results:**

There were 4,741 and 4,734 NLSCYA respondents in the primary analytic sample for serious fighting and assault conviction, respectively. Maternal Head Start exposure was associated with 0.85 times the risk of serious fighting among offspring (95% CI = 0.71, 1.01), with results driven by Black (RR = 0.71, 95% CI = 0.58, 0.87) and Hispanic/Latino male offspring (RR = 0.73, 95% CI = 0.58, 0.92). No reductions in risk of serious fighting were observed among other subpopulations or for assault conviction, a rare outcome.

**Conclusions:**

Results of this study indicate that high-quality early childhood education may narrow disparities in interpersonal violence across generations, offering novel evidence on population-level and primary prevention programs to promote safety and wellbeing.

**Supplementary Information:**

The online version contains supplementary material available at 10.1186/s40621-026-00669-2.

## Background

In 2023, 22,829 Americans died from homicide and many more sustained nonfatal injuries, lived with the threat of violence, and experienced ripple effects of violence, with wide disparities across axes of marginalization and privilege [1]. Black and Brown males are especially disproportionately involved in interpersonal violence, reflecting and reinforcing societal inequities [1, 2]. The social, health, and economic consequences of interpersonal violence in the United States (US) are substantial, with monetary estimates of approximately $200–300 billion per year (in 2025 dollars) for homicide alone [3–5]. 

Efforts to immediately reduce the toll of interpersonal violence in the US (e.g., via intervention with those at highest risk of violence involvement) are critically needed, but long-term, sustained reductions in violence likely require complementary investments in primary prevention strategies that target upstream drivers, i.e., the conditions that put people “at risk of risks,” per the theory of fundamental causes of health inequalities [6, 7]. The underlying causes of interpersonal violence are multifaceted, but many are rooted in social and economic factors and related adversities and opportunities [8]. For example, exposure to poverty and associated stress and trauma—especially early in life—can increase violence risk by shaping socio-emotional and cognitive development (e.g., hypervigilance to threats) and restricting access to flexible resources that provide alternatives to violence (e.g., money, knowledge, power, social capital, neighborhood safety) [6, 9–11]. 

Despite extensive evidence documenting social and economic drivers of interpersonal violence, there is limited research on the violence-prevention effects of interventions that modify upstream social and economic conditions—especially intergenerationally. This is an important gap since present-day violence risk partly reflects “embodied history” [12] (i.e., the cumulative biological, psychosocial, and behavioral effects of social conditions over time and generations), as articulated in the ecosocial theory of disease distribution [13–15]. For example, weathering, the deleterious consequences of accumulated racism-related stressors over time and generations among Black populations [16, 17], has been associated with adverse pregnancy outcomes (e.g., low birth weight) [18] which are, in turn, associated with youth behavioral problems [19]. Further, about a third of Americans who grow up in poverty remain in low income households in adulthood (with notable racial disparities) [20], highlighting one pathway through which economic disadvantage, a key risk factor for violence, can be transmitted across generations. Because interpersonal violence is rooted in part in cumulative intergenerational exposures, interventions that modify social conditions and resources may have intergenerational, accumulating effects.

We examined the long-term impacts of high-quality early childhood education, specifically Head Start, on the next generation’s risk of interpersonal violence. Head Start, launched as part of the War on Poverty, is the nation’s oldest and largest preschool program for low-income children ages 3–5; it incorporates comprehensive education, health, and social services, including support for parents [21]. High-quality early childhood education programs such as Head Start are designed to improve life-long economic, social, and human capital of children and families, and they are generally effective in doing so [22]. For example, research suggests children who attend high-quality early childhood education programs, including Head Start, have improved socio-emotional skills, educational attainment, and employment; higher incomes; and lower risk of aggression, handgun carrying, and crime [23–32]. A handful of studies have examined the intergenerational effects of early childhood education programs [33–39]. All have found meaningful benefits, including for offspring educational attainment, employment, teenage pregnancy, and crime (none examined violent crime) [33–35]. These studies suggest that, to the extent that early childhood interventions such as Head Start improve the life trajectory of one generation, they can disrupt the transmission of disadvantage to the next.

Following prior research, we leveraged the quasi-random rollout of Head Start in the 1960’s and the timing of birth cohorts in the National Longitudinal Survey of Youth 1979 (NLSY79) to examine the intergenerational impacts of mothers’ Head Start exposure on their children’s risk of serious fighting and assault conviction. Drawing on fundamental causes theory, ecosocial theory, and life course perspectives, we hypothesized that maternal Head Start exposure would reduce offsprings’ risk of interpersonal violence by intervening during a sensitive developmental window to shape mothers’ subsequent resources, opportunities, and behaviors—with downstream benefits to their offspring [6, 12, 40]. We also examined heterogeneity in the associations by offspring intersectional social identities (sex, race, and ethnicity), hypothesizing that Black and Hispanic/Latino male offspring would experience the greatest benefits based on prior research showing stronger protective effects for those at greatest risk of adverse outcomes [29, 41–46] and according to resource substitution theory, which suggests marginalized populations benefit more from education because their access to other flexible resources is more restricted [47, 48]. 

## Data & methods

### Study design, setting & population

This retrospective cohort study used intergenerationally linked NLSY79 and Child and Young Adult Cohort (NLSCYA) data. NLSY79 is a nationally representative survey of 12,686 individuals aged 14–21 as of 1978 (born 1957–1964, aged 5 in 1962–1969), and NLSCYA follows the biological children (*n* = 11,551) of female NLSY79 respondents (children of male NLSY79 respondents were not included). Children were born between 1970 and 2014. NLSY79 was administered annually from 1979 to 1994 and biennially through 2020; NLSCYA was administered biennially from 1986 to 2020.

We excluded 1,077 NLSCYA respondents whose mother was born outside the US since exposure was defined by county of birth. For our primary analysis, we then restricted to NLSCYA respondents whose maternal grandmother had less than a high school degree, as in prior work [33], because Head Start is a program for low-income children and families (family income during mothers’ childhood was not measured, education is a salient and commonly used indicator of socioeconomic status, and about two thirds of mothers with children in Head Start in the 1960's had less than a high school degree) [49, 50]. 

### Exposure

Following prior studies [33, 51, 52], the exposure was county-level Head Start availability when mothers were aged 3–5 years induced by variation in Head Start rollout in the 1960’s. We refer to this ecological measure of availability as “maternal Head Start exposure.” Our approach leveraged the Office of Economic Opportunity’s well-documented “wild sort of grant-making operation,” [51, 53] which resulted in Head Start being launched in a somewhat random manner across counties. We used this natural experiment to compare outcomes of children born to mothers of different birth cohorts in counties that launched Head Start at different times or not at all by 1970 (when all mothers were aged 6 + and ineligible), yielding a difference-in-differences comparison. Prior research suggests Head Start participation among low-income children in counties where it was available was between 53–86% [33, 54]. 

We compared outcomes among offspring whose mothers were exposed to Head Start for any amount of time to those whose mothers were never exposed. However, to allow flexibility in model specification (described below), we created a categorical variable representing Head Start exposure “intensity,” defined as the number of years mothers were exposed to Head Start from 3 to 5 years (0 = no exposure, 1 = exposed for 1 year, 2 = exposed for 2 years, and 3 = exposed for 3 years). Data on Head Start rollout were sourced from the National Archives and Records Administration and Head Start program directories and made available by Bailey and colleagues [51, 55, 56]. Data on mothers’ year of birth were obtained from public use NLSY79 files, and data on mothers’ county of birth were obtained from restricted-use NLSY79 geocode files.

### Outcomes

Based on offspring self-report, outcomes were binary indicators for whether NLSCYA respondents ever engaged in serious fighting (i.e., hurt someone badly) from ages 10–17 (measured 1988–2020) and ever reported conviction for assault (“an attack with a weapon or your hands, such as battery, rape, aggravated assault, or manslaughter”) in a survey between ages 15–25 years (measured 1994–2020) (Additional File 1, eTable 1). We excluded 9 and 26 people from the analytic sample for serious fighting and assault conviction, respectively, who were never age-eligible (e.g., excluding those who had not turned 15 by 2020 for assault conviction).

### Modifiers

We considered modification by intersectional social identities (race, ethnicity, and sex). Race and ethnicity were conceptualized as social categories reflecting systematic social (dis)advantage [57]. We used maternal self-reported racial and ethnic origin, per NLSY79, since NLSCYA respondents’ race and ethnicity were not consistently collected. Due to small sample sizes and interpretability, we categorized race and ethnicity as Black, Hispanic/Latino, and White (Additional File 1, eTable 1). Offspring sex (defined as male or female) was measured based on mother report and reconciled (as needed) with information in the child interview [58]. 

### Covariates

Guided by our directed acyclic graph (Additional File 1, eFigure 1), prior research, and available data, we included baseline (1960) characteristics of mothers’ birth county, sourced from the US Census and included in Bailey and colleagues’ replication files [51, 56]: median income, percentage with income less than $3000, percentage aged 25 + years with < 4 years of schooling, log population size, age and racial distribution, rural/urban status, and launch of Community Action Program Health grants prior to 1970 (the only other War on Poverty programs found to be associated with Head Start roll out [33, 51]). At the individual-level, we included the educational attainment of mothers’ father (educational attainment of mothers’ mother was used to define the sample) and whether mothers had an older sibling, spoke a language other than English in childhood, and lived with their biological parents prior to age 3. See Additional File 1, eTable 1 for details.

### Analysis

We used parametric g-computation to estimate average treatment effects on the treated, or how risk of interpersonal violence among the second generation would change had mothers who were exposed to Head Start (for any amount of time) been counterfactually unexposed [59, 60]. We compared outcomes via risk ratios (RR) and differences (RD).

Following Wooldridge, 2021, 2023 [61, 62], we used a flexible logistic regression with indicators for the year mothers’ birth county launched Head Start (reference=never/after 1969), mothers’ birth year, and Head Start exposure (interaction of county launch and birth cohort), allowing coefficients to differ by exposure intensity (see “Model Specification” in Additional File 1). Adjusted models included covariates described above, and all models included two- and three-way interactions between race and ethnicity, sex, and Head Start exposure intensity. As previously described [61, 62], this flexible model avoids problems with more restrictive two-way fixed effect models that impose a single coefficient on a staggered exposure. This approach also integrates seamlessly with g-computation as they both use substitution to estimate marginal contrasts (generating predicted probabilities of the outcome under different exposure settings).

Our difference-in-differences approach assumes that birth cohort effects for offspring of unexposed mothers reflect birth cohort effects that offspring of exposed mothers would have experienced in the absence of Head Start exposure (i.e., parallel trends). We assessed this by plotting event study estimates (Additional File 1, eFigure 2). Following recommended practice [61, 62], we corrected for mild parallel trends violations by incorporating separate linear birth cohort trends (“heterogenous linear trends”) for counties that ever vs. never launched Head Start by 1970. Results without heterogenous linear trends are in Additional File 1, eFigures 3–4.

For modification analyses, we compared outcomes among subpopulations defined by intersectional social identities (without setting these social identities to counterfactual values) [63]. 

We censored respondents if they were never interviewed during the outcome measurement window. Censoring was considered another intervention (i.e., we estimated the joint effect of hypothetically removing maternal Head Start exposure for those exposed *and* preventing censoring throughout follow-up [64]); thus, estimates reflect comparisons had everyone remained uncensored. Missing data were multiply imputed (see “Multiple Imputation” in Additional File 1). We used a clustered bootstrap (*n* = 200) for inference (clustering by NLSY79 primary sampling units and strata), combining bootstrap results using Rubin’s rules [65]. Following prior research, we did not incorporate survey weights [66]. We did not report results if estimates were highly uncertain (e.g., confidence limit ratio of 30 or higher).

Analyses were conducted in R version 4.1.1 (R Foundation for Statistical Computing, Vienna, Austria). NLSY79 and NLSCYA were approved by the Institutional Review Boards (IRB) at the Ohio State University and National Opinion Research Center at the University of Chicago [67]. The University of Washington IRB considered this study not human subjects research since data were deidentified.

### Sensitivity analyses

As a falsification test, we limited the sample to NLSCYA respondents whose maternal grandmother had a high school degree or higher. Since this group was less likely eligible for Head Start (we estimated that, in 1960, about 11% of women aged 25 + with a high school degree or higher and children under 5 were in poverty, versus nearly 40% of those with less than a high school degree [68]), we conceptualized them as a negative control population [69]. That is, we expected this population to be exposed to other county-level changes that shaped community health and wellbeing (i.e., confounders) but be unexposed to Head Start (as they were largely ineligible to participate). If we observed an association between Head Start and outcomes among the negative control population, it would suggest that other, co-occurring county-level factors helped explain results observed in our primary study population (NLSCYA respondents whose maternal grandmother had less than a high school degree). If we did not observe an association among the negative control population, it would suggest our primary results were not confounded by other county-level changes.

## Results

### Study population

There were 4,741 NLSCYA respondents in the primary analytic sample for serious fighting and 4,734 NLSCYA respondents in the primary analytic sample for assault conviction. For both samples, approximately 30% of mothers were exposed to Head Start (i.e., lived in a county in which Head Start was available) at some point when they were aged 3–5 years (Table 1). About 50% of offspring were male and one-third were Black. Overall, approximately 26–32% of offspring reported serious fighting (35–40% among those who remained uncensored), and 3.5–3.9% reported assault conviction (4.8–5.1% among those who remained uncensored). Additional File 1, eTables 2, 3 and 4 describe the study population by intersectional social identities.

**Table 1:** Description of NLSCYA Respondents Whose Maternal Grandmother Had Less Than a High School Degree

	Serious fighting analytic sample			Assault conviction analytic sample		
	Total, *N* = 4,741	Mother exposed to Head Start, *N* = 1,456	Mother not exposed to Head Start, *N* = 2,655	Total, *N* = 4,734	Mother exposed to Head Start, *N* = 1,452	Mother not exposed to Head Start, *N* = 2,653
	No. (%)	No. (%)	No. (%)	No. (%)	No. (%)	No. (%)
**Offspring**						
Race and ethnicity^a^						
American Indian, Alaska Native	575 (12.1%)	143 (9.8%)	359 (13.5%)	575 (12.1%)	143 (9.8%)	359 (13.5%)
Asian	51 (1.1%)	21 (1.4%)	22 (0.8%)	51 (1.1%)	21 (1.4%)	22 (0.8%)
Black	1,675 (35.3%)	461 (31.7%)	973 (36.6%)	1,672 (35.3%)	460 (31.7%)	972 (36.6%)
Another race	578 (12.2%)	169 (11.6%)	373 (14.0%)	577 (12.2%)	168 (11.6%)	373 (14.1%)
White	1,097 (23.1%)	356 (24. 5%)	660 (24.9%)	1,095 (23.1%)	354 (24.4%)	660 (24.9%)
Hispanic ethnicity	1,027 (21.7%)	397 (27.3%)	405 (15.3%)	1,026 (21.7%)	397 (27. 3%)	404 (15.2%)
Sex						
Male	2,385 (50.3%)	737 (50.6%)	1,316 (49.6%)	2,380 (50.3%)	734 (50.6%)	1,314 (49.5%)
Female	2,355 (49.7%)	719 (49.4%)	1,338 (50.4%)	2,353 (49.7%)	718 (49.4%)	1,338 (50.4%)
Serious fighting (10–17)						
No	2,158 (45.5%)	670 (46.0%)	1,217 (45.8%)	NA	NA	NA
Yes	1,354 (28.6%)	470 (32.3%)	700 (26.4%)	NA	NA	NA
Censored^b^	1,067 (22.5%)	265 (18.2%)	655 (24.7%)	NA	NA	NA
Assault conviction (15–25)						
No	NA	NA	NA	3,320 (70.1%)	1,117 (76.9%)	1,766 (66.6%)
Yes	NA	NA	NA	179 (3.8%)	56 (3.9%)	93 (3.5%)
Censored^b^	NA	NA	NA	1,222 (25.8%)	275 (18.9%)	785 (29.6%)
**Mother baseline**						
*Individual-level*						
Mother foreign language in childhood						
No	3,496 (73.7%)	1,000 (68.7%)	2,133 (80.3%)	3,490 (73.7%)	996 (68.6%)	2,132 (80.4%)
Yes	1,234 (26.0%)	451 (31.0%)	516 (19.4%)	1,233 (26.0%)	451 (31.1%)	515 (19.4%)
Mother older sibling						
No	801 (16.9%)	210 (14.4%)	476 (17.9%)	801 (16.9%)	210 (14.5%)	476 (17.9%)
Yes	3,799 (80.1%)	1,212 (83.2%)	2,096 (78.9%)	3,792 (80.1%)	1,208 (83.2%)	2,094 (78.9%)
Mother’s father’s education						
Less than high school	2,732 (57.6%)	835 (57.3%)	1,612 (60.7%)	2,726 (57.6%)	831 (57.2%)	1,610 (60.7%)
At least high school	1,056 (22.3%)	358 (24.6%)	484 (18.2%)	1,055 (22.3%)	358 (24.7%)	484 (18.2%)
Lived with both biological parents prior to age 3						
No	656 (13.8%)	213 (14.6%)	332 (12.5%)	657 (13.9%)	213 (14.7%)	333 (12.6%)
Yes	3,642 (76.8%)	1,146 (78.7%)	2,056 (77.4%)	3,635 (76.8%)	1,143 (78.7%)	2,053 (77.4%)
Mother birth year, mean (SD)	1,960.60 (41.4)	1,962.54 (134.8)	1,959.48 (73.8)	1,960.59 (41.4)	1,962.54 (135.2)	1,959.48 (73.9)
*Mother’s County of birth*						
Year first Head Start grant						
By 1969	3,294 (69.5%)	1,456 (100%)	1,838 (69.2%)	3,290 (69.5%)	1,452 (100%)	1,838 (69.3%)
After 1969 or never	817 (17.2%)	0 (0%)	817 (30.8%)	815 (17.2%)	0 (0%)	815 (30.7%)
Years mother exposed to Head Start						
0	2,655 (56%)	0 (0%)	2,655 (100%)	2,653 (56.0%)	0 (0%)	2,653 (100%)
1	437 (9.2%)	437 (30.0%)	0 (0%)	436 (9.2%)	436 (30.0%)	0 (0%)
2	434 (9.2%)	434 (29.8%)	0 (0%)	433 (9.1%)	433 (29.8%)	0 (0%)
3	585 (12.3%)	585 (40.2%)	0 (0%)	583 (12.3%)	583 (40.2%)	0 (0%)
CAP Health grant prior to 1970						
No	1,924 (40.6%)	357 (24.5%)	1,567 (59.0%)	1,922 (40.6%)	356 (24.5%)	1,566 (59.0%)
Yes	2,215 (46.7%)	1,099 (75.5%)	1,088 (41.0%)	2,211 (46.7%)	1,096 (75.5%)	1,087 (41.0%)
Log county population (1960), mean (SD)	11.92 (1.70)	12.29 (1.34)	11.37 (1.48)	11.92 (1.70)	12.29 (1.34)	11.37 (1.48)
County percent urban (1960), mean (SD)	64 (31)	72 (24)	55 (31)	64 (31)	72 (24)	55 (31)
County percent non-white (1960), mean (SD)	16 (17)	15 (15)	18 (19)	16 (17)	15 (15)	18 (19)
County percent aged 0–4 years (1960), mean (SD)	11.83 (1.71)	12.01 (1.67)	11.98 (1.69)	11.83 (1.71)	12.02 (1.67)	11.98 (1.69)
County median family income in dollars (1959), mean (SD)	4,988 (1,554)	5,208 (1,385)	4,605 (1,540)	4,988 (1,554)	5,206 (1,386)	4,607 (1,539)
County percent income <$3000 (1959), mean (SD)	28 (17)	26 (15)	32 (17)	28 (17)	26 (15)	32 (17)
County percent < 4 years schooling (1960), mean (SD)	13 (9)	12 (9)	14 (9)	13 (9)	13 (9)	14 (9)

NA = not applicable; CAP = Community Action Program; SD = standard deviation

Rows and columns may not sum to total because of missing values

^a^Based on maternal race and ethnicity reported in NLSY79 (see Additional File 1, eTable 1 for details). The “another race” category includes response options “other” and “American.” We considered the response option “none” as missing. Counts for Native Hawaiian and Pacific Islander offspring are not shown because of suppression rules (per restricted-use geocode data). Per NLSY, the prevalence of the category Indian American, or Native American is unusually high, perhaps because respondents misinterpreted the term “Native American”

^b^Individuals were censored if they were eligible but never interviewed during the outcome measurement window

### Serious fighting

**Fig. 1:**
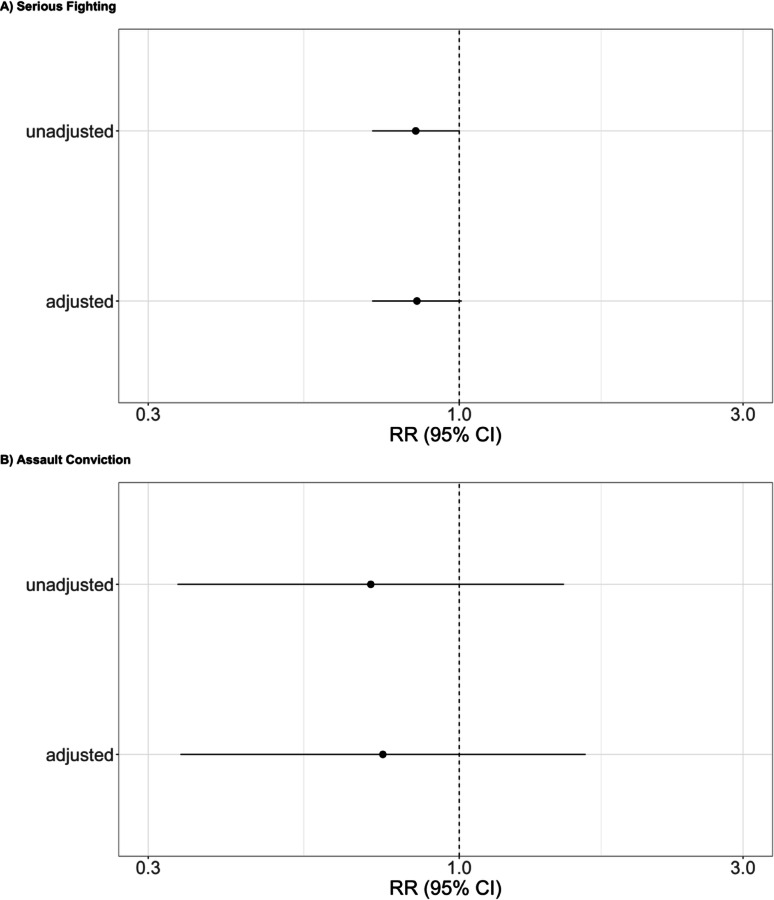
Relative Risk of Offspring Violence Associated Maternal Head Start Exposure Among NLSCYA Respondents Whose Maternal Grandmother Had Less Than a High School Degree. Adjusted estimates control for variables described in the text. All estimates control for heterogenous linear birth cohort trends for county Head Start adoption status

**Fig. 2:**
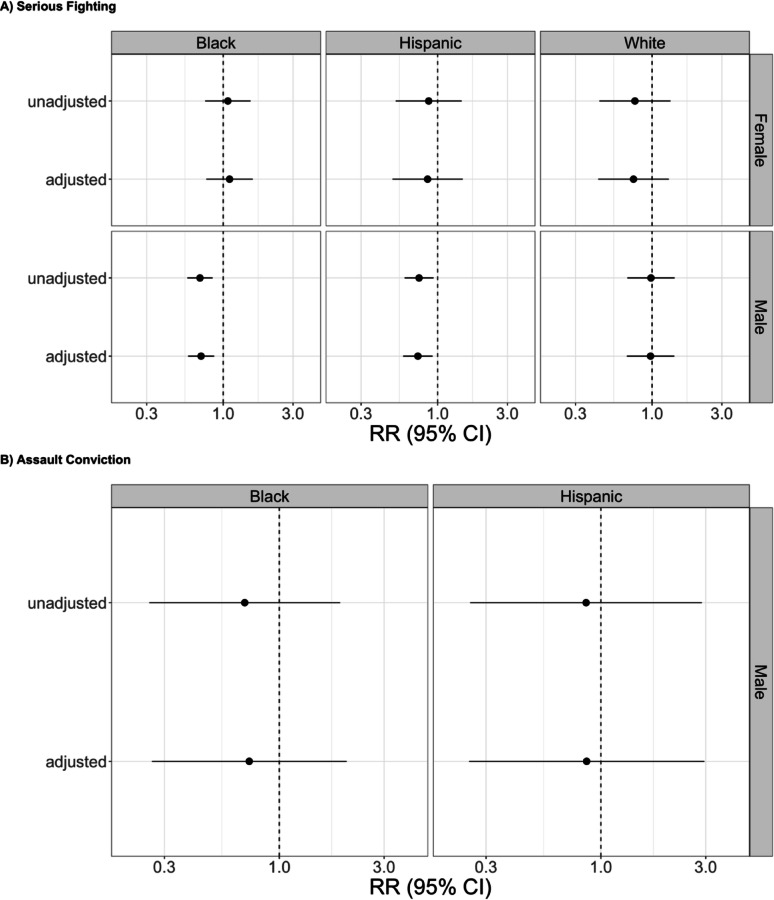
Relative Risk of Offspring Violence Associated Maternal Head Start Exposure Among NLSCYA Respondents Whose Maternal Grandmother Had Less Than a High School Degree, By Intersectional Social Identities. Results not shown if the confidence limit ratio was 30 or higher. Adjusted estimates control for variables described in the text. All estimates control for heterogenous linear birth cohort trends for county Head Start adoption status

Offspring whose mother was exposed to Head Start had an estimated 15.1% lower risk of serious fighting than they would have had if their mother was not exposed to Head Start (RR = 0.85, 95% CI = 0.71, 1.01, Fig. 1A; RD=-7.2% points, 95% CI=-16.1%, 1.7% points, Additional File 1, eFigure 5 A), with results driven by Black male offspring (RR = 0.71, 95% CI = 0.58, 0.87, Fig. 2A; RD=-20.8% points, 95% CI=-34.9%, -6.7% points, Additional File 1, eFigure 6 A) and Hispanic/Latino male offspring (RR = 0.73, 95% CI = 0.58, 0.92, Fig. 2A; RD=-16.7% points, 95% CI=-33.2%, -0.2% points, Additional File 1, eFigure 6 A). Estimates for other intersectional groups were not distinguishable from the null.

### Assault conviction

Estimates for the association of maternal Head Start exposure and offspring assault conviction were imprecise and not distinguishable from the null, overall (Fig. 1B; Additional File 1, eFigure 5B) and among intersectional groups (Fig. 2B; Additional File 1, eFigure 6B).

### Sensitivity analyses

When limiting to NLSCYA respondents whose maternal grandmother had a high school degree or higher (falsification test), estimates for serious fighting among Black and Hispanic/Latino males became attenuated and not distinguishable from the null (Additional File 1, eFigures 7–8), supporting findings from our primary analysis.

## Discussion

This study assessed the intergenerational impacts of mothers’ exposure to Head Start—a large-scale, high-quality early childhood education program for low-income children—on their offspring’s risk of interpersonal violence in adolescence and early adulthood. We estimated that Black and Hispanic/Latino males whose mother lived a county that launched Head Start by the time she was age eligible (3–5 years old) had meaningfully lower risk of serious fighting than they would have had if their mother had lived in a county that did not launch Head Start by the time she was eligible. There was no reduction in the risk of serious fighting for other subpopulations or for assault conviction, a rare outcome in our sample. Because Black and Hispanic/Latino males are disproportionately likely to be involved in violence, these findings indicate that Head Start may narrow disparities in serious fighting—an important outcome itself and a potential indicator for and/or precursor to other significant forms of harm (e.g., homicide) [70, 71]. 

Our findings of protective associations align with prior intergenerational research on early childhood education and other outcomes. For example, Barr and Gibbs, 2022 found that maternal Head Start exposure was associated with lower risk of teenage parenthood and crime (defined as any arrest, conviction, or probation) among offspring and increased likelihood of offspring graduating high school, attending college, and earning higher wages [33]. In that study, estimates for several outcomes were larger for male and Black offspring, but estimates for crime were larger for non-Black offspring (19.7% point reduction) than Black offspring (6.1% point reduction). García et al., 2021—whose sample was entirely comprised of Black individuals—found that offspring of Perry Preschool Program participants experienced benefits in various outcomes, including school suspension, health, employment, stable relationships, and arrest [34]. They found that effect estimates were typically larger for males than females (e.g., the largest reductions in risk of arrest were among male offspring of male program participants) [34], consistent with evidence that boys are more sensitive to family disadvantage and thus experience greater returns from intervention [72]. 

The rarity of assault conviction and our relatively small sample size could explain why we found no association of maternal Head Start exposure and offspring’s risk of assault conviction. It could also be that gendered and racialized disparities in police surveillance and the criminal legal system [73] resulted in a higher likelihood of arrest, charge, and conviction for males of color (approximately 6–8% of Black and Hispanic/Latino male offspring in our study were convicted of assault, compared with less than 2% of White male offspring), thus limiting our ability to detect effects of Head Start. Other ongoing structural barriers (e.g., in employment, housing, education systems) could also limit the protection conferred by Head Start, especially over long time horizons [74]. Additional work is needed to understand factors that mediate and modify the intergenerational impact of early childhood education on the next generation’s risk of violent crime.

Overall, our findings add novel evidence on the potential intergenerational benefits of early childhood education at a time when continued federal support for Head Start is uncertain. For example, recently, some Head Start classrooms closed temporarily because they had not received federal funds, several Head Start offices closed after layoffs at the Department of Health and Human Services, the Administration proposed millions in cuts to Head Start (which did not pass), and the Department of Health and Human Services reclassified Head Start to make it inaccessible to immigrants (a directive currently being challenged in court) [75–79]. Our results, along with prior evidence, suggest such disinvestments and restrictions on access may create long-term harm, and, instead, access to high-quality early childhood education should be expanded. Indeed, prior cost-benefit analyses suggest Head Start produces returns on investment [80], especially relative to its reasonable costs: in 2023, the entire program cost approximately $12 billion, or 0.7% of federal discretionary spending [81], and research estimates that for every dollar invested in Head Start, from $1.50 to $2.66 is saved [82, 83]. Our findings add new evidence to inform future cost-benefit analyses. Interpersonal violence has wide-ranging individual, community, and societal costs. For example, in 2020, the total estimated economic burden of youth interpersonal violence in the US (including medical spending, lost work productivity, reduced quality of life from injury, and avoidable mortality) was $122 billion [84]. Nationally, the annual cost of school violent crime (based on data spanning 1993–2016) was estimated to be almost $200 million (including counseling, work loss, medical costs, and property damage) [85], and a study of Texas public schools from 2008 to 2009 estimated that the state’s 1030 districts each spent an average of $312 thousand on security measures alone [86]. Primary prevention of interpersonal violence can reduce such financial burdens (and their opportunity costs) and improve individual and population health in various other non-enumerable ways.

### Limitations

Although we leveraged quasi-random rollout of Head Start and conducted robustness checks, there may still be residual confounding. We did not have data on Head Start attendance, so estimates reflect the potential impacts of Head Start rollout on offspring of participants and non-participants in counties in which it was available. There may be misclassification of exposure if mother’s birth county differed from their county of residence during ages 3–5 years, or if they were exposed to Migrant and Seasonal Head Start, which began in 1969. We lacked detailed data and sufficient sample size to examine more granular racial and ethnic groups. Race and ethnicity for offspring were also assigned based on mothers’ self-reported racial and ethnic origins; thus, while the sample only included biological offspring, there may be misclassification (e.g., multiracial offspring may report their race and ethnicity differently). Further, like all survey research, there may be measurement error (e.g., social desirability or recall bias). We focused on self-reported assault conviction, and results may have differed if we had used administrative criminal records. While self-reported criminal convictions may have some bias (e.g., social desirability), they also have advantages over administrative records, which can underestimate criminal involvement (e.g., because no centralized national database exists on criminal records) [87]. NLSCYA did not ask questions about assault conviction of those in jail or prison at the time of the survey prior to 2006, so assault conviction may be underestimated for individuals whose entire outcome measurement window was prior to 2006, potentially also contributing to null results for assault conviction. Finally, offspring of male NLSY79 respondents were not included, and findings may not generalize to paternal Head Start exposure.

## Conclusion

This national longitudinal study suggests that maternal Head Start exposure meaningfully reduced risk of serious fighting in adolescence and early adulthood among Black and Hispanic/Latino male offspring, indicating that high-quality early childhood education may narrow intergenerational disparities in interpersonal violence. Findings underscore the potential for policies that invest in young children and families to address enduring social and structural drivers of violence across generations.

## Supplementary Information

Supplementary Material 1

## Data Availability

The datasets generated and/or analysed during the current study are available in the National Longitudinal Survey data repository, (https:/www.nlsinfo.org) . Geocode data are available from the US Bureau of Labor Statistics but restrictions apply to the availability of these data, which were used under license for the current study, and so are not publicly available.

## Abbreviations

NLSY79: National Longitudinal Survey of Youth 1979
NLSCYA: National Longitudinal Survey Child and Young Adult Cohort
US: United States
RR: Risk ratio
RD: Risk difference
