# Supplementary material for: Intergenerational association of early childhood education and interpersonal violence: a retrospective cohort study
Source: Inj Epidemiol. 2026 Mar 13;13:28. doi: 10.1186/s40621-026-00669-2 (PMC13097770; doi:10.1186/s40621-026-00669-2)
Supplement: Supplementary file 1 — Supplementary Material 1 [file 40621_2026_669_MOESM1_ESM.docx]

**Additional File 1**

Intergenerational association of early childhood education and interpersonal violence: A retrospective cohort study

Julia P. Schleimer, PhD, MPH,^a,b^ Anjum Hajat, PhD, MPH,^a^ Gail Joseph, PhD,^c^ Min Sun, PhD,^c^ Frederick Rivara, MD, MPH,^a,b^Ali Rowhani-Rahbar, MD, PhD, MPH^a,b^

^a^Department of Epidemiology, School of Public Health, University of Washington, Seattle, WA, USA

^b^Center for Firearm Injury Prevention, Department of Pediatrics, School of Medicine, University of Washington, Seattle, WA, USA

^c^College of Education, University of Washington, Seattle, WA, USA

Table of Contents

[eFigure 1. Directed Acyclic Graph 3](#_Toc223026676)

[eTable 1. Definition of Variables 5](#_Toc223026677)

[Model Specification 12](#_Toc223026678)

[Multiple Imputation 14](#_Toc223026679)

[eTable 2. Description of Black NLSCYA Respondents Whose Maternal Grandmother Had Less Than a High School Degree 16](#_Toc223026680)

[eTable 3. Description of Hispanic/Latino NLSCYA Respondents Whose Maternal Grandmother Had Less Than a High School Degree 19](#_Toc223026681)

[eTable 4. Description of White NLSCYA Respondents Whose Maternal Grandmother Had Less Than a High School Degree 22](#_Toc223026682)

[eFigure 2. Event Study Estimates of the Relative Risk of Offspring Violence Associated with Maternal Head Start Exposure Among NLSCYA Respondents Whose Maternal Grandmother Had Less Than a High School Degree 25](#_Toc223026683)

[eFigure 3. Relative Risk of Offspring Violence Associated with Maternal Head Start Exposure Among NLSCYA Respondents Whose Maternal Grandmother Had Less Than a High School Degree, *Without Heterogenous Linear Trends* 27](#_Toc223026684)

[eFigure 4. Relative Risk of Offspring Violence Associated with Maternal Head Start Exposure Among NLSCYA Respondents Whose Maternal Grandmother Had Less Than a High School Degree, by Intersectional Social Identities, *Without Heterogenous Linear Trends* 28](#_Toc223026685)

[eFigure 5. Difference in Risk of Offspring Violence Associated with Maternal Head Start Exposure Among NLSCYA Respondents Whose Maternal Grandmother Had Less Than a High School Degree *(Additive Scale)* 29](#_Toc223026686)

[eFigure 6. Difference in Risk of Offspring Violence Associated with Maternal Head Start Exposure Among NLSCYA Respondents Whose Maternal Grandmother Had Less Than a High School Degree, By Intersectional Social Identities *(Additive Scale)* 30](#_Toc223026687)

[eFigure 7. Relative Risk of Offspring Serious Fighting Associated with Maternal Head Start Exposure Among NLSCYA Respondents Whose Maternal Grandmother Had a *High School Degree or Higher (Falsification Test)* 31](#_Toc223026688)

[eFigure 8. Relative Risk of Offspring Serious Fighting Associated with Maternal Head Start Exposure Among NLSCYA Respondents Whose Maternal Grandmother Had a *High School Degree or Higher (Falsification Test)*, By Intersectional Social Identities 32](#_Toc223026689)

[References 33](#_Toc223026690)

### eFigure 1. Directed Acyclic Graph


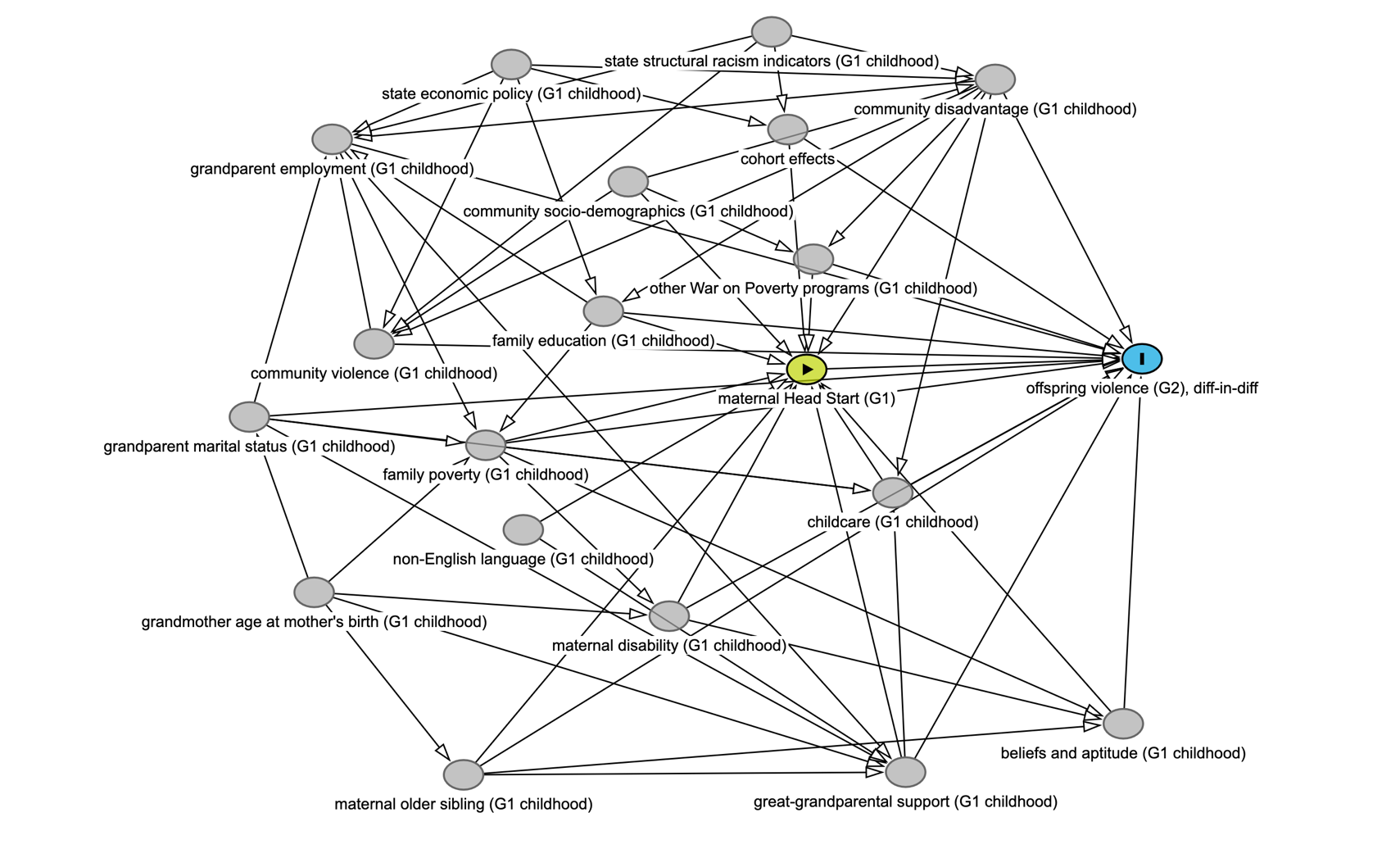


G1 = generation 1 (mothers), G2 = generation 2 (offspring), diff-in-diff = difference-in-differences

Minimally sufficient adjustment set to control for confounding of maternal Head Start exposure and outcomes of offspring violence:

- cohort effects
- community disadvantage (G1 childhood)
- community population & urbanicity (G1 childhood)
- family education (G1 childhood)
- other War on Poverty programs (G1 childhood)
- maternal older sibling (G1 childhood)
- non-English language (G1 childhood)
- beliefs and aptitude (G1 childhood)
- childcare (G1 childhood)
- family poverty (G1 childhood)
- great-grandparental support (G1 childhood)
- maternal disability (G1 childhood)

Blue text indicates unmeasured confounders. However, childcare (G1 childhood) was proxied by whether mothers lived with both biological parents during early childhood. Because we used a difference-in-differences analysis, confounder will only bias the associations under study if they differentially affected mothers born in Head Start counties when Head Start was launched (i.e., stable characteristics of counties and common birth cohort trends are differenced out).

### eTable 1. Definition of Variables

| **Variable** | **Definition** | **Source and notes** | **Analytic operationalization** |
| --- | --- | --- | --- |
| **Offspring** | | | |
| Race and ethnicity | Maternal race and ethnicity (used as proxy for offspring race and ethnicity) | Obtained from NLSY79 in first survey wave (1979)  Questions: “What is your origin or descent?” with response options (select all that apply): Black, Chinese, English, Filipino, French, German, Greek, Hawaiian or Pacific Islander, Indian-American or Native American, Asian Indian, Irish, Italian, Japanese, Korean, Cuban, Chicano, Mexican, Mexican-American, Puerto Rican, other Hispanic, other Spanish, Polish, Portuguese, Russian, Scottish, Vietnamese, Welsh, other, American. And: “You said that your origin or descent was (categories coded in prior question). Which one of these do you feel closest to?” with the same response options as above.  We first categorized race as American Indian Alaska Native; Asian (Chinese, Filipino, Asian Indian, Japanese, Korean, Vietnamese); Black; Native Hawaiian or Pacific Islander; other race (none, other, American); White (English, French, German, Greek, Irish, Italian, Polish, Portuguese, Russian, Scottish, Welsh); and Hispanic or Latino (Cuban, Chicano, Mexican, Mexican-American, Puerto Rican, other Hispanic, other Spanish). Categories were based on those used by the Office of Budget and Management.^1^  For analyses, due to sample size and interpretability, we then coded individuals as Black if they reported Black as their origin; Hispanic/Latino if they reported Hispanic/Latino as their origin; and White if they reported White (as defined above) as their *only* or *primary* origin and did not report any other race or ethnicity (excluding American Indian or Alaska Native due to measurement issues as noted by NLSY^2^). | Set of separate binary indicators for Black, Hispanic or Latino, and White |
| Sex | Offspring sex | Obtained from NLSCYA, “The official fertility record in the mother's main Youth record is reconciled with reports in the child interview to ascertain the best determination of the child's sex.”^3^ | Binary variable (male, female) |
| Serious Fighting | Ever hurt someone bad enough to need bandages or a doctor from ages 10-17 years | Question: “In the last year, about how many times have you hurt someone bad enough to need bandages or a doctor?” with response options never, once, twice, and more than twice, and “In the last year (last 12 months), have you ever hurt someone badly enough to need bandages or a doctor?” with response options yes and no. Questions were asked in the child survey of those 10 and older in 1988-1992 and 10-14 in 1994-2014, and questions were asked in a separate young adult survey of those 15-17 in 1994-2012 and 12-17 in 2014-2020. | Binary variable (yes, no) |
| Assault conviction | Ever reported ever being convicted of assault in a survey round between ages 15-25 years | Questions: “Have you ever been convicted of any charges other than a minor traffic violation?” with response options yes and no. “What charges have you been convicted of? (circle all that apply)” and “What charges were you most recently convicted of?” We focused on the response option “Assault (an attack with a weapon or your hands, such as battery, rape, aggravated assault, or manslaughter).” From 2006-2020, questions were asked of those in jail or prison at the time of the survey. | Binary variable (yes, no) |
| **Mother baseline** | | | |
| *Individual-level* | | | |
| Spoke a language other than English in childhood | Whether mother spoke a language other than English at home when she was a child | Obtained from NLSY79 in first survey wave (1979)  Question: “when you were a child, was any language, other than English, spoken in your home?” with response options: yes, no | Binary variable (yes, no) |
| Mother older sibling | Whether mother had an older sibling | Obtained from NLSY79 in first survey wave (1979)  Respondents were asked the number of siblings they had and then “how many of your brothers and sisters are older than you?” with number response options, which we recoded as any or none | Binary variable (yes, no) |
| Educational attainment of mother’s father | Mother’s father’s highest grade or year of schooling | Obtained from NLSY79 in first survey wave (1979)  Question: “What is the highest grade or year of regular school that your father ever completed?” with response options: none, 1^st^ grade, 2^nd^ grade, 3^rd^ grade, 4^th^ grade, 5^th^ grade, 6^th^ grade, 7^th^ grade, 8^th^ grade, 9^th^ grade, 10^th^ grade, 11^th^ grade, 12^th^ grade, 1^st^ year college, 2^nd^ year college, 3^rd^ year college, 4^th^ year college, 5^th^ year college, 6^th^ year college, 7^th^ year college, 8^th^ year college or more | Categorical variable (less than high school, high school, more than high school) |
| Educational attainment of mother’s mother | Mother’s mother’s highest grade or year of schooling | Obtained from NLSY79 in first survey wave (1979)  Question: “What is the highest grade or year of regular school that your mother ever completed?” with response options: none, 1^st^ grade, 2^nd^ grade, 3^rd^ grade, 4^th^ grade, 5^th^ grade, 6^th^ grade, 7^th^ grade, 8^th^ grade, 9^th^ grade, 10^th^ grade, 11^th^ grade, 12^th^ grade, 1^st^ year college, 2^nd^ year college, 3^rd^ year college, 4^th^ year college, 5^th^ year college, 6^th^ year college, 7^th^ year college, 8^th^ year college or more | Categorical variable (less than high school, high school, more than high school) |
| Mother lived with biological parents prior to age 3 | Whether mother lived with both biological parents prior to age 3 | Obtained from NLSY79 in first survey wave (1979)  Question: “did you live with both your biological mother and biological father from the time you were born until your 18th birthday?” with response options: yes, no. Those who did not live with both biological parents from birth to age 18 were asked: “At what ages did you live with your biological mother? We will only record situations which lasted more than four months. (Probe:) What other times?” with response options for never, birth, age 1, age 2, and age 3; and “At what ages did you live with your biological father? We will only record situations which lasted more than four months. (Probe:) What other times?” with response options for never, birth, age 1, age 2, and age 3.  Based on the answers to these questions, we created categories for whether mother lived with neither/one, or both biological parents prior to age 3. | Binary variable (yes no) |
| **Mother’s county of birth** | | | |
| Head Start exposure | Year mother’s birth county launched Head Start | Obtained from Bailey et al., 2021 and 2015 replication file^4,5^  As in prior work,^4,6^ we considered 1966 the first Head Start launch year, as funding awarded in 1965 was largely recorded in fiscal year 1966.  Based on mother’s birth year and the year her birth county launched Head Start, we coded mothers as being exposed (i.e., lived in a county with Head Start) for 0, 1, 2, or 3 years from ages 3-5. | Categorical variable (number of years of exposure: 0, 1, 2, 3) |
| Community Action Program (CAP) grants | Whether mothers’ county of birth had a CAP health grant program by 1970 | Obtained from Bailey et al., 2021 and 2015 replication file^4,5^ | Binary variable (yes, no) |
| County population size | Total county population for mothers’ county of birth, 1960 | Obtained from Bailey et al., 2021 and 2015 replication file^4,5^ | Log continuous variable |
| County rural/urban status | Percentage of the county population living in urban area for mothers’ county of birth, 1960 | Obtained from Bailey et al., 2021 and 2015 replication file^4,5^ | Continuous variable |
| County non-White | Percentage of the county population that was non-White for mothers’ county of birth, 1960 | Obtained from Bailey et al., 2021 and 2015 replication file^4,5^ | Continuous variable |
| County % age 0-4 | Percentage of the county population aged 0-4 years for mothers’ county of birth, 1960 | Obtained from Bailey et al., 2021 and 2015 replication file^4,5^ | Continuous variable |
| County median income | Median family income for mothers’ county of birth, 1959 | Obtained from Bailey et al., 2021 and 2015 replication file^4,5^ | Continuous variable |
| County % income < $3000 | Percentage of the county population with income less than $3000 in mothers’ county of birth, 1959 | Obtained from Bailey et al., 2021 and 2015 replication file^4,5^ | Continuous variable |
| County % < 4 years of schooling | Percentage of the county population aged 25+ with less than 4 years of schooling in mothers’ county of birth, 1960 | Obtained from Bailey et al., 2021 and 2015 replication file^4,5^ | Continuous variable |

### Model Specification

$$logit\left( y_{jit} \right)= \alpha+ {\beta_{1}c}_{i}+ {\beta_{2}f}_{t}+{\sum_{k=1}^{p} \beta_{1k}r}_{jk}+{\beta_{3}s}_{j}+\sum_{k=1}^{p} \beta_{2k}\left( r_{jk}*s_{j} \right)+\beta_{4}\left( w_{it}*n_{it} \right)+\sum_{k=1}^{p} \beta_{3k}\left( w_{it}*n_{it}*r_{jk} \right)+\beta_{5}\left( w_{it}*n_{it}*s_{j} \right)+\sum_{k=1}^{p} \beta_{4k}\left( w_{it}*n_{it}*r_{jk}*s_{j} \right)+{\sum_{k=1}^{p} \beta_{5k}X}_{ik}+{\sum_{k=1}^{p} \beta_{6k}Z}_{jk}+ \sum_{k=1}^{p} \beta_{7k}\left( t_{t}*r_{jk} \right)+\beta_{6}\left( t_{t}*s_{j} \right)+\sum_{k=1}^{p} \beta_{8k}\left( t_{t}*r_{jk}*s_{j} \right)+\sum_{k=1}^{p} \beta_{9k}\left( l_{i}*r_{jk} \right)+\beta_{7}\left( l_{i}*s_{j} \right)+\sum_{k=1}^{p} \beta_{10k}\left( l_{i}*r_{jk}*s_{j} \right)+\beta_{8}\left( t_{t}*l_{i} \right)+\sum_{k=1}^{p} \beta_{11k}\left( t_{t}*l_{i}*r_{jk} \right)+\beta_{9}\left( t_{t}*l_{i}*s_{j} \right)+\sum_{k=1}^{p} \beta_{12k}\left( t_{t}*l_{i}*r_{jk}*s_{j} \right)$$

- $y_{jit}$ is the outcome among NLSCYA respondent $j$ whose mother was born in county $i$ and year $t$
- $\alpha$ is the model intercept
- $c_{i}$ are county cohort fixed effects (i.e., cohorts defined by year of Head Start launch, with never/after 1969 as the reference)
- $f_{t}$ are birth cohort fixed effects (i.e., mothers’ year of birth)
- $r_{j}$ are separate indicators for race and ethnicity, with k indexing variables (Black yes/no, Hispanic or Latino yes/no, and White yes/no)
- $s_{j}$ is an indicator for offspring sex (female, male)
- $w_{it}$ is a binary indicator for any maternal Head Start exposure (0, 1) reflecting the interaction of mothers’ county cohort and birth year. Although redundant with $n_{it}$ (defined below), this term is included to facilitate marginal predictions under exposed vs. unexposed scenarios
- $n_{it}$ is a categorical variable for the number of years mothers were exposed to Head Start during age 3-5 years (0, 1, 2, or 3 years, i.e., exposure “intensity”, with 0 as the reference) based on the interaction of mothers’ county cohort and birth year
- $X_{i}$ indicates a vector of baseline county-level covariates, with k indexing variables (characterizing mothers’ county of birth)
- $Z_{j}$ indicates a vector of baseline individual-level covariates, with k indexing variables (characterizing mothers’ childhood)
- $t_{t}$ is a linear time trend for maternal birth year
- $l_{i}$ is an indicator for whether mother’s birth county launched Head Start by 1970 (yes/no)
- $\beta_{4},\beta_{3k},\beta_{5},\beta_{4k}$ reflect ATTs, which we aggregated via marginal contrasts (i.e., predicting outcomes when $w_{it}$ was 0 and 1 and taking the average contrast, either ratio or difference, among those who were in fact exposed)
- Note: Unadjusted models excluded $X_{i}$ and $Z_{j}$, and models without linear birth cohort trends excluded $\beta_{8},\beta_{11k},\beta_{9}, \beta_{12k}$

Our analysis considered the joint intervention of receiving maternal Head Start exposure and removing censoring, as described by Hernán and Robins (Chapter 8).^7^ Because removing censoring is considered another intervention (in additional to removing Head Start exposure for those exposed), we need to account for confounding between censoring and the outcomes.^7^ The primary confounder between censoring and outcomes is whether offsprings’ mother was in a certain NLSY79 oversample (military oversample or economically-disadvantaged non-Black and non-Hispanic oversample), since NLSY removed these groups from follow-up, but the defining characteristics of these dropped oversamples may be related to outcomes. Further, because the likelihood of a mother being in a dropped oversample (and thus outcomes for their offspring censored) might be a consequence of Head Start exposure (i.e., if Head Start affected mother’s income or military participation), this confounder is also a potential mediator of Head Start’s effect on offspring interpersonal violence. To account for such time-dependent confounding, we controlled for being in a dropped oversample (yes vs. no) via the iterated conditional expectation (ICE) implementation of g-computation.^8,9^ This involved first fitting a regression model as specified above but additionally controlling for being in a dropped oversample, predicting the outcome, and then fitting a second regression model (as specified above), using the fitted value from the first regression as the outcome for the second.

### Multiple Imputation

We used the mice R package to impute missing data in the exposure, outcomes, and covariates with parametric multiple imputation including interactions. Imputation models included NLSY79 cluster and strata variables to account for the complex sampling design.^10^ We imputed 10 datasets, conducted the analysis (including bootstrapping) on each imputed data, and pooled results using Rubin’s rules. We chose variables to include in our imputation model because a) they were used in our inferential analyses for this study or related studies, b) we hypothesized them to be predictive of the exposure or outcome, or c) they characterized the complex survey design. Specifically, we included the following variables in our imputation model:

- Head Start exposure (number of years exposed to Head Start during ages 3-5 based on county and year of birth), categorical variable (0, 1, 2, 3)
- Mother’s birth year, categorical variable
- Year of Head Start launch in mother’s county of birth, categorical variable
- Offspring ever reported serious fighting from ages 10-17, binary variable
- Offspring ever convicted of assault from ages 15-25, binary variable
- Offspring ever reported suicidal ideation from age 15+, binary variable
- Offspring ever reported depression from age 15+, binary variable
- Log of population in mother’s county of birth, continuous variable
- Year of first Community Action Program Planning grant in mother’s county of birth, continuous variable
- Year of first Community Action Program Health grant in mother’s county of birth, continuous variable
- Year of first Community Action Program Seniors grant in mother’s county of birth, continuous variable
- Year of first Legal Services Program grant in mother’s county of birth, continuous variable
- Year of first Food Stamp grant in mother’s county of birth, continuous variable
- Year of first Community Health Center grant in mother’s county of birth, continuous variable
- Percent urban in mother’s county of birth, continuous
- Percent rural farm in mother’s county of birth, continuous
- Percent non-White in mother’s county of birth, continuous
- Percent aged 0-4 years in mother’s county of birth, continuous
- Percent aged 65 and older in mother’s county of birth, continuous
- Median family income mother’s county of birth, continuous
- Percent income less than $3000 in mother’s county of birth, continuous
- Percent income $10,000 and higher in mother’s county of birth, continuous
- Median years schooling in mother’s county of birth, continuous
- Percent less than 4 years of schooling in mother’s county of birth, continuous
- Percent less than 12 years of schooling in mother’s county of birth, continuous
- Language other than English spoken at home during mother’s childhood, binary variable
- Mother lived with neither/one or both biological parents prior to age 3, binary variable
- Mother had older sibling, binary variable
- Mother’s mother’s highest educational attainment, categorical variable (less than high school, high school, more than high school)
- Mother’s father’s highest educational attainment, categorical variable (less than high school, high school, more than high school)
- Either of mother’s parents foreign born, binary variable
- Mother’s poverty status during ages 17-22 years, binary variable
- Mother’s marital status during ages 17-22 years, binary variable
- Whether mother lived with a parent during ages 17-22 years, binary variable
- Average proportion of weeks mother was unemployed during ages 17-22 years, continuous variable
- Whether mother had completed high school or GED before age 23, binary variable
- Year of offspring birth, continuous variable
- Average value of Black-White dissimilarity index in mother’s county of residence during ages 17-22 years, continuous variable
- Average homicide rate in mother’s county of residence during ages 17-22 years, continuous variable
- Average socioeconomic disadvantage in mother’s county of residence during ages 17-22 years, continuous variable
- Average value of labor scale in mother’s state of residence during ages 17-22 years, continuous variable
- Average value of criminal legal system and voting scales in mother’s state of residence during ages 17-22 years, continuous variable
- Race and ethnicity (Black, Hispanic or Latino, White), binary variables
- Offspring sex, binary variable
- Two and three-way interactions between Head Start exposure and race, ethnicity, sex
- Two and three-way interactions between race, ethnicity, sex and county and state-level variables (county homicide rate, county disadvantage, state labor policies, and state criminal legal system and voting policies)
- NLSY79 primary sampling units and strata

### eTable 2. Description of Black NLSCYA Respondents Whose Maternal Grandmother Had Less Than a High School Degree

|  | Serious fighting analytic sample | | | | Assault conviction analytic sample | | | |
| --- | --- | --- | --- | --- | --- | --- | --- | --- |
|  | Mother exposed to Head Start | | Mother not exposed to Head Start | | Mother exposed to Head Start | | Mother not exposed to Head Start | |
| Offspring sex | Male, n = 235 | Female, n = 226 | Male, n = 458 | Female, n = 515 | Male, n = 234 | Female, n = 226 | Male, n = 456 | Female, n = 516 |
|  | No. (%) | No. (%) | No. (%) | No. (%) | No. (%) | No. (%) | No. (%) | No. (%) |
| **Offspring** |  |  |  |  |  |  |  |  |
| Serious fighting (10-17) |  |  |  |  |  |  |  |  |
| No | 103 (44%) | 126 (56%) | 188 (41%) | 315 (61%) | NA | NA | NA | NA |
| Yes | 103 (44%) | -- | 199 (43%) | 132 (26%) | NA | NA | NA | NA |
| Censored^a^ | 24 (10%) | -- | 61 (13%) | 57 (11%) | NA | NA | NA | NA |
| Assault conviction (15-25) |  |  |  |  |  |  |  |  |
| No | NA | NA | NA | NA | 188 (80%) | 203 (90%) | 360 (79%) | 437 (85%) |
| Yes | NA | NA | NA | NA | 18 (7.7%) | -- | 31 (6.8%) | 15 (2.9%) |
| Censored^a^ | NA | NA | NA | NA | 25 (11%) | -- | 61 (13%) | 63 (12%) |
| **Mother baseline** |  |  |  |  |  |  |  |  |
| *Individual-level* |  |  |  |  |  |  |  |  |
| Mother foreign language in childhood |  |  |  |  |  |  |  |  |
| No | -- | -- | 446 (97%) | -- | -- | -- | 444 (97%) | -- |
| Yes | -- | -- | 11 (2.4%) | -- | -- | -- | 11 (2.4%) | -- |
| Mother older sibling |  |  |  |  |  |  |  |  |
| No | 28 (12%) | 19 (8.4%) | 73 (16%) | 74 (14%) | 28 (12%) | 19 (8.4%) | 73 (16%) | 74 (14%) |
| Yes | 201 (86%) | 203 (90%) | 378 (83%) | 430 (83%) | 200 (85%) | 203 (90%) | 376 (82%) | 431 (84%) |
| Mother's father's education |  |  |  |  |  |  |  |  |
| Less than high school | 117 (50%) | 110 (49%) | 279 (61%) | 304 (59%) | 116 (50%) | 110 (49%) | 277 (61%) | 305 (59%) |
| At least high school | 53 (23%) | 54 (24%) | 40 (8.7%) | 51 (9.9%) | 53 (23%) | 54 (24%) | 40 (8.8%) | 51 (9.9%) |
| Lived with both biological parents prior to age 3 |  |  |  |  |  |  |  |  |
| No | 72 (31%) | 56 (25%) | 105 (23%) | 119 (23%) | 72 (31%) | 56 (25%) | 105 (23%) | 120 (23%) |
| Yes | 149 (63%) | 162 (72%) | 310 (68%) | 338 (66%) | 148 (63%) | 162 (72%) | 308 (68%) | 338 (66%) |
| Mother birth year | 1,962.52 (1.09) | 1,962.61 (0.99) | 1,959.63 (1.94) | 1,959.66 (1.88) | 1,962.52 (1.09) | 1,962.61 (0.99) | 1,959.62 (1.94) | 1,959.66 (1.88) |
| *Mother’s county of birth* |  |  |  |  |  |  |  |  |
| Year first Head Start grant |  |  |  |  |  |  |  |  |
| By 1969 | 235 (100%) | 226 (100%) | 285 (62%) | 322 (63%) | 234 (100%) | 226 (100%) | 284 (62%) | 323 (63%) |
| After 1969 or never | 0 (0%) | 0 (0%) | 173 (38%) | 193 (37%) | 0 (0%) | 0 (0%) | 172 (38%) | 193 (37%) |
| Years mother exposed to Head Start |  |  |  |  |  |  |  |  |
| 1 | 66 (28%) | 52 (23%) | NA | NA | 66 (28%) | 52 (23%) | NA | NA |
| 2 | 78 (33%) | 74 (33%) | NA | NA | 77 (33%) | 74 (33%) | NA | NA |
| 3 | 91 (39%) | 100 (44%) | NA | NA | 91 (39%) | 100 (44%) | NA | NA |
| CAP Health grant prior to 1970 |  |  |  |  |  |  |  |  |
| No | 56 (24%) | 49 (22%) | 290 (63%) | 325 (63%) | 56 (24%) | 49 (22%) | 289 (63%) | 326 (63%) |
| Yes | 179 (76%) | 177 (78%) | 168 (37%) | 190 (37%) | 178 (76%) | 177 (78%) | 167 (37%) | 190 (37%) |
| Log county population (1960), mean (SD) | 12.39 (1.48) | 12.18 (1.52) | 11.26 (1.56) | 11.27 (1.58) | 12.39 (1.48) | 12.18 (1.52) | 11.26 (1.56) | 11.27 (1.58) |
| County percent urban (1960), mean (SD) | 72 (28) | 68 (30) | 48 (34) | 49 (34) | 72 (28) | 68 (30) | 48 (34) | 49 (34) |
| County percent non-white (1960), mean (SD) | 26 (16) | 28 (18) | 33 (19) | 34 (19) | 26 (16) | 28 (18) | 33 (19) | 34 (19) |
| County percent aged 0-4 years (1960), mean (SD) | 11.92 (1.44) | 11.99 (1.57) | 12.21 (1.56) | 12.23 (1.63) | 11.93 (1.44) | 11.99 (1.57) | 12.21 (1.57) | 12.23 (1.63) |
| County median family income (1959), mean (SD) | 4,900 (1,595) | 4,666 (1,718) | 4,009 (1,541) | 3,994 (1,622) | 4,899 (1,598) | 4,666 (1,718) | 4,013 (1,543) | 3,995 (1,620) |
| County percent income <$3000 (1959), mean (SD) | 29 (18) | 33 (19) | 38 (18) | 39 (18) | 30 (18) | 33 (19) | 38 (18) | 39 (18) |
| County percent <4 years schooling (1960), mean (SD) | 13 (8) | 15 (9) | 17 (8) | 18 (9) | 13 (8) | 15 (9) | 17 (8) | 18 (9) |

NA = not applicable; CAP = Community Action Program; SD = standard deviation

Rows and columns may not sum to total because of missing values

-- indicates small cell counts (<=10) are suppressed/redacted to mitigate risk of disclosure for restricted-use geocode information

^a^Individuals were censored if they were eligible but never interviewed during the outcome measurement window

### eTable 3. Description of Hispanic/Latino NLSCYA Respondents Whose Maternal Grandmother Had Less Than a High School Degree

|  | Serious fighting analytic sample | | | | Assault conviction analytic sample | | | |
| --- | --- | --- | --- | --- | --- | --- | --- | --- |
|  | Mother exposed to Head Start | | Mother not exposed to Head Start | | Mother exposed to Head Start | | Mother not exposed to Head Start | |
| Offspring sex | Male, n = 201 | Female, n = 196 | Male, n = 212 | Female, n = 193 | Male, n = 201 | Female, n = 196 | Male, n = 212 | Female, n = 192 |
|  | No. (%) | No. (%) | No. (%) | No. (%) | No. (%) | No. (%) | No. (%) | No. (%) |
| **Offspring** |  |  |  |  |  |  |  |  |
| Serious fighting (10-17) |  |  |  |  |  |  |  |  |
| No | 90 (45%) | 114 (58%) | 91 (43%) | 128 (66%) | NA | NA | NA | NA |
| Yes | 79 (39%) | 53 (27%) | 87 (41%) | 38 (20%) | NA | NA | NA | NA |
| Censored^a^ | 22 (11%) | 23 (12%) | 21 (9.9%) | 23 (12%) | NA | NA | NA | NA |
| Assault conviction (15-25) |  |  |  |  |  |  |  |  |
| No | NA | NA | NA | NA | 166 (83%) | 171 (87%) | 175 (83%) | 163 (85%) |
| Yes | NA | NA | NA | NA | -- | -- | 14 (6.6%) | -- |
| Censored^a^ | NA | NA | NA | NA | -- | -- | 23 (11%) | -- |
| **Mother baseline** |  |  |  |  |  |  |  |  |
| *Individual-level* |  |  |  |  |  |  |  |  |
| Mother foreign language in childhood |  |  |  |  |  |  |  |  |
| No | 12 (6.0%) | 13 (6.6%) | 15 (7.1%) | -- | 12 (6.0%) | 13 (6.6%) | 15 (7.1%) | -- |
| Yes | 189 (94%) | 183 (93%) | 197 (93%) | -- | 189 (94%) | 183 (93%) | 197 (93%) | -- |
| Mother older sibling |  |  |  |  |  |  |  |  |
| No | 25 (12%) | 31 (16%) | 32 (15%) | 40 (21%) | 25 (12%) | 31 (16%) | 32 (15%) | 40 (21%) |
| Yes | 172 (86%) | 159 (81%) | 170 (80%) | 147 (76%) | 172 (86%) | 159 (81%) | 170 (80%) | 146 (76%) |
| Mother's father's education |  |  |  |  |  |  |  |  |
| Less than high school | 131 (65%) | 131 (67%) | 147 (69%) | 20 (10%) | 131 (65%) | 131 (67%) | 147 (69%) | 137 (71%) |
| At least high school | 36 (18%) | 34 (17%) | 21 (9.9%) | 138 (72%) | 36 (18%) | 34 (17%) | 21 (9.9%) | 20 (10%) |
| Lived with both biological parents prior to age 3 |  |  |  |  |  |  |  |  |
| No | 27 (13%) | 23 (12%) | -- | -- | 27 (13%) | 23 (12%) | -- | -- |
| Yes | 160 (80%) | 158 (81%) | -- | -- | 160 (80%) | 158 (81%) | -- | -- |
| Mother birth year | 1,962.50 (1.11) | 1,962.57 (1.11) | 1,959.42 (1.71) | 1,959.04 (1.62) | 1,962.50 (1.11) | 1,962.57 (1.11) | 1,959.42 (1.71) | 1,959.02 (1.60) |
| *Mother’s county of birth* |  |  |  |  |  |  |  |  |
| Year first Head Start grant |  |  |  |  |  |  |  |  |
| By 1969 | 201 (100%) | 196 (100%) | 193 (91%) | 180 (93%) | 201 (100%) | 196 (100%) | 193 (91%) | 180 (94%) |
| After 1969 or never | 0 (0%) | 0 (0%) | 19 (9.0%) | 13 (6.7%) | 0 (0%) | 0 (0%) | 19 (9.0%) | 12 (6.2%) |
| Years mother exposed to Head Start |  |  |  |  |  |  |  |  |
| 1 | 61 (30%) | 59 (30%) | NA | NA | 61 (30%) | 59 (30%) | NA | NA |
| 2 | 62 (31%) | 52 (27%) | NA | NA | 62 (31%) | 52 (27%) | NA | NA |
| 3 | 78 (39%) | 85 (43%) | NA | NA | 78 (39%) | 85 (43%) | NA | NA |
| CAP Health grant prior to 1970 |  |  |  |  |  |  |  |  |
| No | 45 (22%) | 46 (23%) | 88 (42%) | 76 (39%) | 45 (22%) | 46 (23%) | 88 (42%) | 75 (39%) |
| Yes | 156 (78%) | 150 (77%) | 124 (58%) | 117 (61%) | 156 (78%) | 150 (77%) | 124 (58%) | 117 (61%) |
| Log county population (1960), mean (SD) | 12.35 (1.19) | 12.23 (1.23) | 11.74 (1.43) | 11.90 (1.33) | 12.35 (1.19) | 12.23 (1.23) | 11.74 (1.43) | 11.91 (1.33) |
| County percent urban (1960), mean (SD) | 77 (18) | 76 (18) | 69 (23) | 71 (22) | 77 (18) | 76 (18) | 69 (23) | 71 (22) |
| County percent non-white (1960), mean (SD) | 5.9 (5.0) | 6.5 (7.5) | 6 (7) | 7 (8) | 5.9 (5.0) | 6.5 (7.5) | 6 (7) | 6 (8) |
| County percent aged 0-4 years (1960), mean (SD) | 12.96 (1.84) | 13.02 (1.88) | 13.06 (1.83) | 12.95 (1.84) | 12.96 (1.84) | 13.02 (1.88) | 13.06 (1.83) | 12.98 (1.82) |
| County median family income (1959), mean (SD) | 5,158 (1,163) | 5,111 (1,163) | 5,025 (1,370) | 5,055 (1,357) | 5,158 (1,163) | 5,111 (1,163) | 5,025 (1,370) | 5,071 (1,341) |
| County percent income <$3000 (1959), mean (SD) | 26 (12) | 26 (12) | 27 (14) | 27 (14) | 26 (12) | 26 (12) | 27 (14) | 27 (14) |
| County percent <4 years schooling (1960), mean (SD) | 16 (12) | 16 (12) | 16 (13) | 16 (12) | 16 (12) | 16 (12) | 16 (13) | 16 (12) |

NA = not applicable; CAP = Community Action Program; SD = standard deviation

Rows and columns may not sum to total because of missing values

-- indicates small cell counts (<=10) are suppressed/redacted to mitigate risk of disclosure for restricted-use geocode information

^a^Individuals were censored if they were eligible but never interviewed during the outcome measurement window

### eTable 4. Description of White NLSCYA Respondents Whose Maternal Grandmother Had Less Than a High School Degree

|  | Serious fighting analytic sample | | | | Assault conviction analytic sample | | | |
| --- | --- | --- | --- | --- | --- | --- | --- | --- |
|  | Mother exposed to Head Start | | Mother not exposed to Head Start | | Mother exposed to Head Start | | Mother not exposed to Head Start | |
| Offspring sex | Male, n = 188 | Female, n = 168 | Male, n = 341 | Female, n = 318 | Male, n = 187 | Female, n = 167 | Male, n = 341 | Female, n = 318 |
|  | No. (%) | No. (%) | No. (%) | No. (%) | No. (%) | No. (%) | No. (%) | No. (%) |
| **Offspring** |  |  |  |  |  |  |  |  |
| Serious fighting (10-17) |  |  |  |  |  |  |  |  |
| No | 67 (36%) | 75 (45%) | 116 (34%) | 145 (46%) | NA | NA | NA | NA |
| Yes | 61 (32%) | 34 (20%) | 80 (23%) | 41 (13%) | NA | NA | NA | NA |
| Censored^a^ | 55 (29%) | 50 (30%) | 135 (40%) | 124 (39%) | NA | NA | NA | NA |
| Assault conviction (15-25) |  |  |  |  |  |  |  |  |
| No | NA | NA | NA | NA | 119 (64%) | 119 (71%) | 167 (49%) | 158 (50%) |
| Yes | NA | NA | NA | NA | -- | -- | -- | -- |
| Censored^a^ | NA | NA | NA | NA | -- | -- | -- | -- |
| **Mother baseline** |  |  |  |  |  |  |  |  |
| *Individual-level* |  |  |  |  |  |  |  |  |
| Mother foreign language in childhood |  |  |  |  |  |  |  |  |
| No | 171 (91%) | 142 (85%) | 294 (86%) | 283 (89%) | 170 (91%) | 141 (84%) | 294 (86%) | 283 (89%) |
| Yes | 16 (8.5%) | 22 (13%) | 46 (13%) | 32 (10%) | 16 (8.6%) | 22 (13%) | 46 (13%) | 32 (10%) |
| Mother older sibling |  |  |  |  |  |  |  |  |
| No | 33 (18%) | 27 (16%) | 74 (22%) | 56 (18%) | 33 (18%) | 27 (16%) | 74 (22%) | 56 (18%) |
| Yes | 151 (80%) | 131 (78%) | 253 (74%) | 248 (78%) | 150 (80%) | 130 (78%) | 253 (74%) | 248 (78%) |
| Mother's father's education |  |  |  |  |  |  |  |  |
| Less than high school | 108 (57%) | 98 (58%) | 201 (59%) | 190 (60%) | 107 (57%) | 97 (58%) | 201 (59%) | 190 (60%) |
| At least high school | 64 (34%) | 53 (32%) | 89 (26%) | 86 (27%) | 64 (34%) | 53 (32%) | 89 (26%) | 86 (27%) |
| Lived with both biological parents prior to age 3 |  |  |  |  |  |  |  |  |
| No | 15 (8.0%) | -- | 30 (8.8%) | 31 (9.7%) | 15 (8.0%) | -- | 30 (8.8%) | 31 (9.7%) |
| Yes | 162 (86%) | -- | 276 (81%) | 253 (80%) | 161 (86%) | -- | 276 (81%) | 253 (80%) |
| Mother birth year | 1,962.48 (1.12) | 1,962.36 (1.10) | 1,959.32 (1.83) | 1,959.52 (1.75) | 1,962.48 (1.11) | 1,962.35 (1.10) | 1,959.32 (1.83) | 1,959.52 (1.75) |
| *Mother’s county of birth* |  |  |  |  |  |  |  |  |
| Year first Head Start grant |  |  |  |  |  |  |  |  |
| By 1969 | 188 (100%) | 168 (100%) | 236 (69%) | 225 (71%) | 187 (100%) | 167 (100%) | 236 (69%) | 225 (71%) |
| After 1969 or never | 0 (0%) | 0 (0%) | 105 (31%) | 93 (29%) | 0 (0%) | 0 (0%) | 105 (31%) | 93 (29%) |
| Years mother exposed to Head Start |  |  |  |  |  |  |  |  |
| 1 | 66 (35%) | 59 (35%) | NA | NA | 66 (35%) | 59 (35%) | NA | NA |
| 2 | 48 (26%) | 55 (33%) | NA | NA | 48 (26%) | 55 (33%) | NA | NA |
| 3 | 74 (39%) | 54 (32%) | NA | NA | 73 (39%) | 53 (32%) | NA | NA |
| CAP Health grant prior to 1970 |  |  |  |  |  |  |  |  |
| No | 46 (24%) | 41 (24%) | 209 (61%) | 187 (59%) | 46 (25%) | 41 (25%) | 209 (61%) | 187 (59%) |
| Yes | 142 (76%) | 127 (76%) | 132 (39%) | 131 (41%) | 141 (75%) | 126 (75%) | 132 (39%) | 131 (41%) |
| Log county population (1960), mean (SD) | 12.34 (1.36) | 12.52 (1.35) | 11.46 (1.40) | 11.52 (1.39) | 12.34 (1.36) | 12.51 (1.35) | 11.46 (1.40) | 11.52 (1.39) |
| County percent urban (1960), mean (SD) | 71 (24) | 75 (22) | 57 (28) | 59 (28) | 71 (24) | 75 (22) | 57 (28) | 59 (28) |
| County percent non-white (1960), mean (SD) | 11 (11) | 11 (11) | 10 (14) | 11 (14) | 11 (11) | 11 (11) | 10 (14) | 11 (14) |
| County percent aged 0-4 years (1960), mean (SD) | 11.05 (1.13) | 11.28 (1.38) | 11.33 (1.42) | 11.50 (1.46) | 11.05 (1.13) | 11.28 (1.38) | 11.33 (1.42) | 11.50 (1.46) |
| County median family income (1959), mean (SD) | 5,546 (1,174) | 5,716 (1,118) | 5,052 (1,376) | 5,032 (1,447) | 5,540 (1,174) | 5,710 (1,118) | 5,052 (1,376) | 5,032 (1,447) |
| County percent income <$3000 (1959), mean (SD) | 22 (11) | 20 (10) | 26 (15) | 27 (15) | 22 (11) | 20 (10) | 26 (15) | 27 (15) |
| County percent <4 years schooling (1960), mean (SD) | 8.8 (4.5) | 8.6 (4.5) | 10 (7) | 10 (7) | 8.8 (4.5) | 8.6 (4.5) | 10 (7) | 10 (7) |

NA = not applicable; CAP = Community Action Program; SD = standard deviation

Rows and columns may not sum to total because of missing values

-- indicates small cell counts (<=10) are suppressed/redacted to mitigate risk of disclosure for restricted-use geocode information

^a^Individuals were censored if they were eligible but never interviewed during the outcome measurement window. Many White offspring were censored because the survey removed children of the economically disadvantaged White NLSY79 oversample in 1990; we considered censoring another intervention, so estimates of the association between Head Start and outcomes reflect comparisons had everyone remained uncensored during follow-up.

### eFigure 2. Event Study Estimates of the Relative Risk of Offspring Violence Associated with Maternal Head Start Exposure Among NLSCYA Respondents Whose Maternal Grandmother Had Less Than a High School Degree

**A) Serious Fighting**


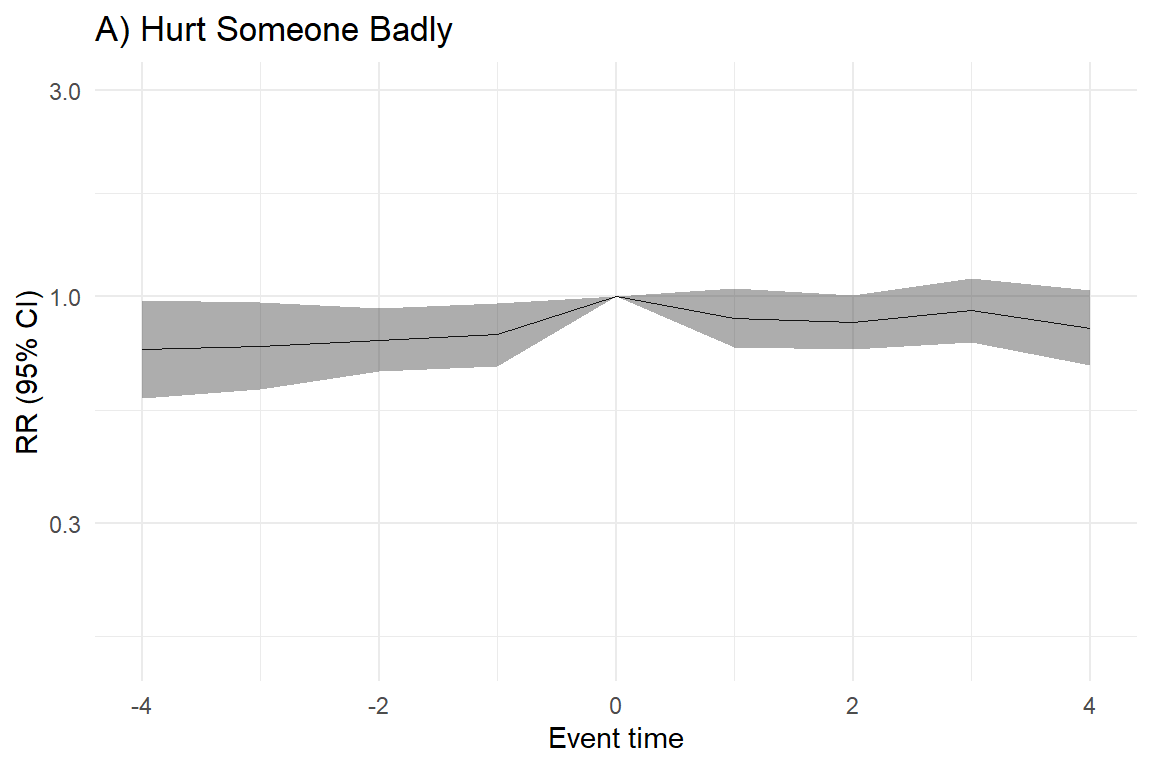


**B) Assault Conviction**


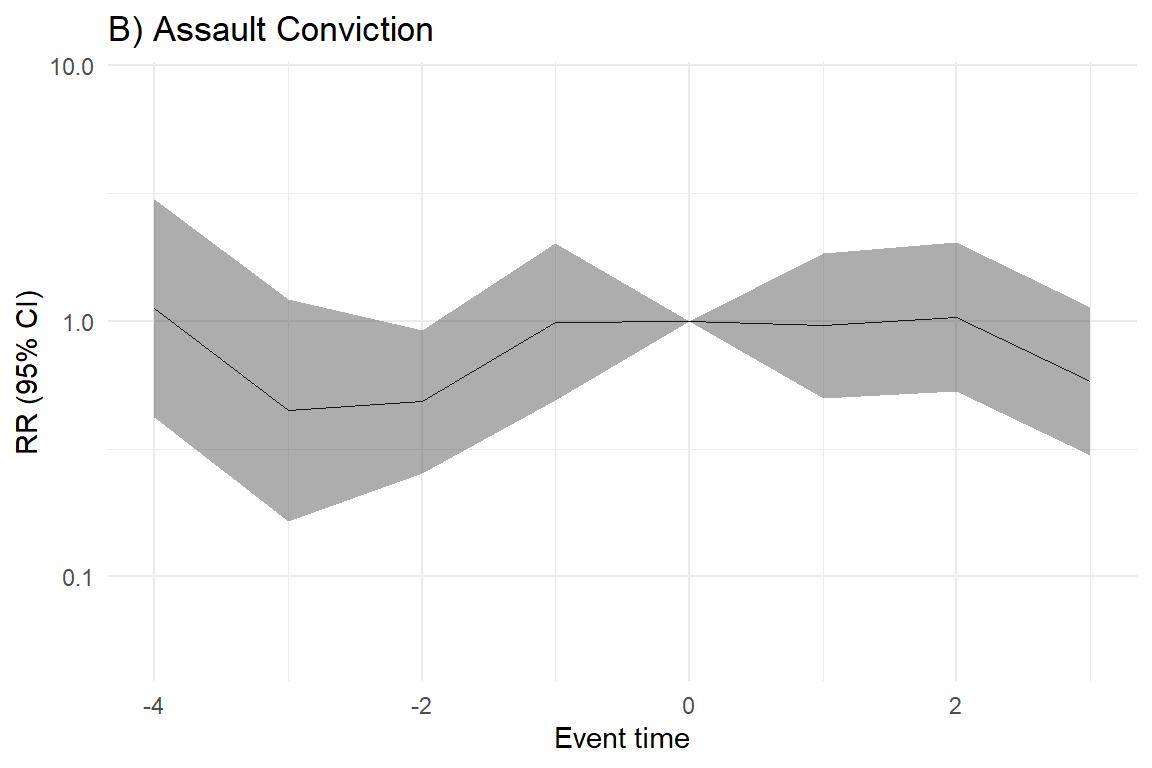


Estimates control for variables described in the text. Time 4 is not shown for assault conviction because the estimate was highly imprecise. These event study estimates suggest offspring of mothers in counties that eventually launched Head Start had slightly higher risk of serious fighting in successive birth cohorts prior to Head Start exposure, relative to offspring whose mothers were born in counties that never launched Head Start by 1970. Thus, failing to adjust for heterogenous trends thus resulted in estimates that were biased upward, as seen in eFigures 3-4.

### eFigure 3. Relative Risk of Offspring Violence Associated with Maternal Head Start Exposure Among NLSCYA Respondents Whose Maternal Grandmother Had Less Than a High School Degree, *Without Heterogenous Linear Trends*


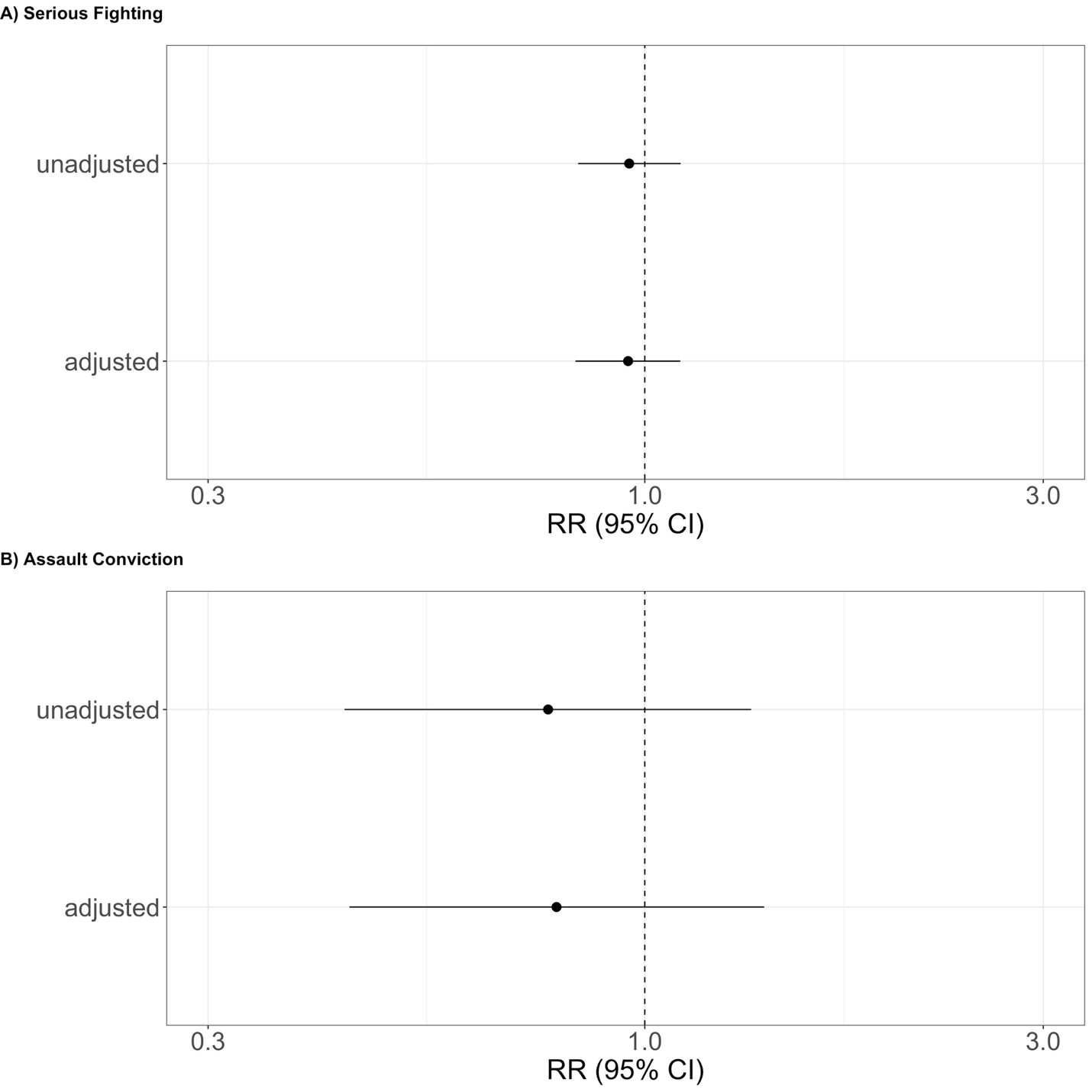


Adjusted estimates control for variables described in the text.

### eFigure 4. Relative Risk of Offspring Violence Associated with Maternal Head Start Exposure Among NLSCYA Respondents Whose Maternal Grandmother Had Less Than a High School Degree, by Intersectional Social Identities, *Without Heterogenous Linear Trends*

*
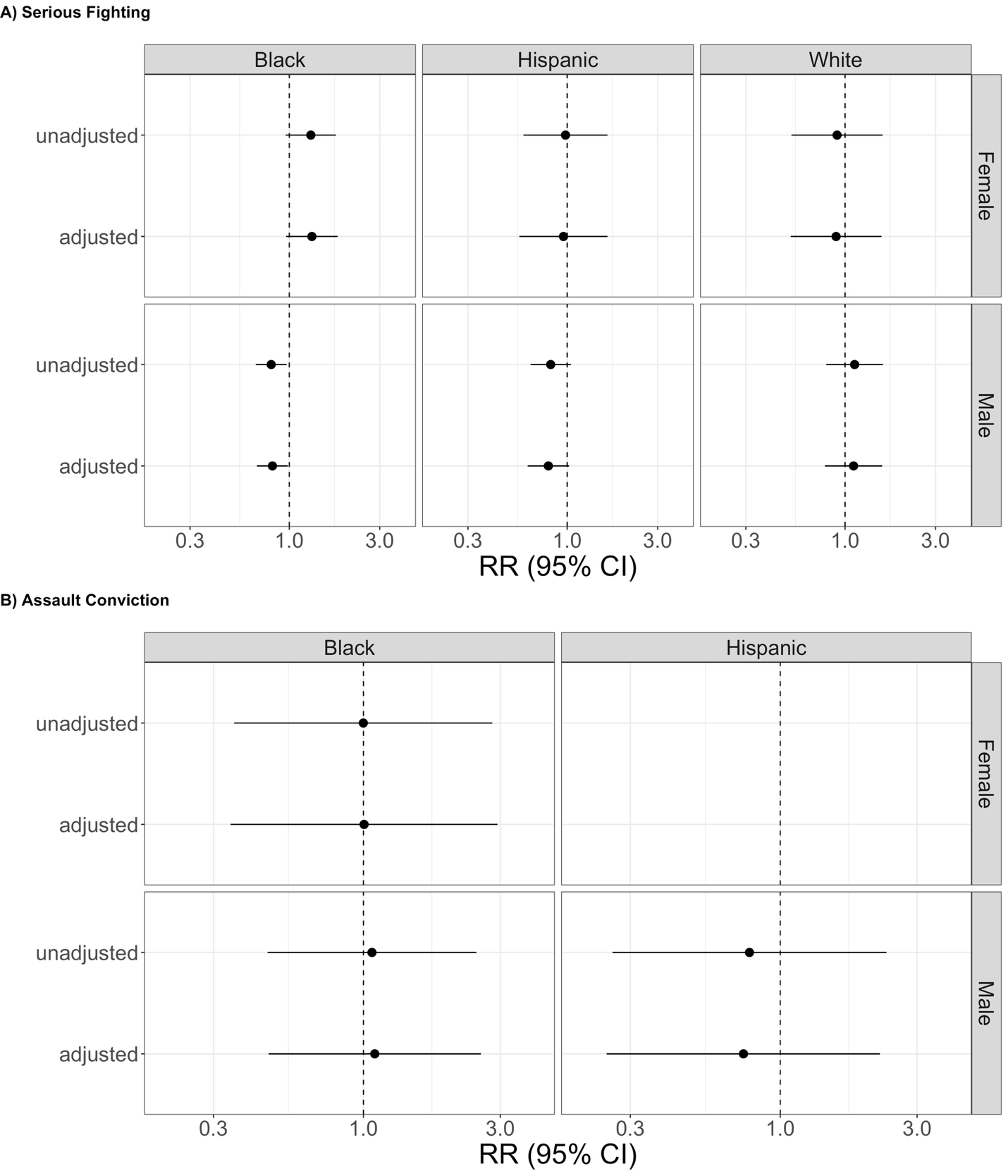
*

Results not shown if the confidence limit ratio was 30 or higher. Adjusted estimates control for variables described in the text.

### eFigure 5. Difference in Risk of Offspring Violence Associated with Maternal Head Start Exposure Among NLSCYA Respondents Whose Maternal Grandmother Had Less Than a High School Degree *(Additive Scale)*


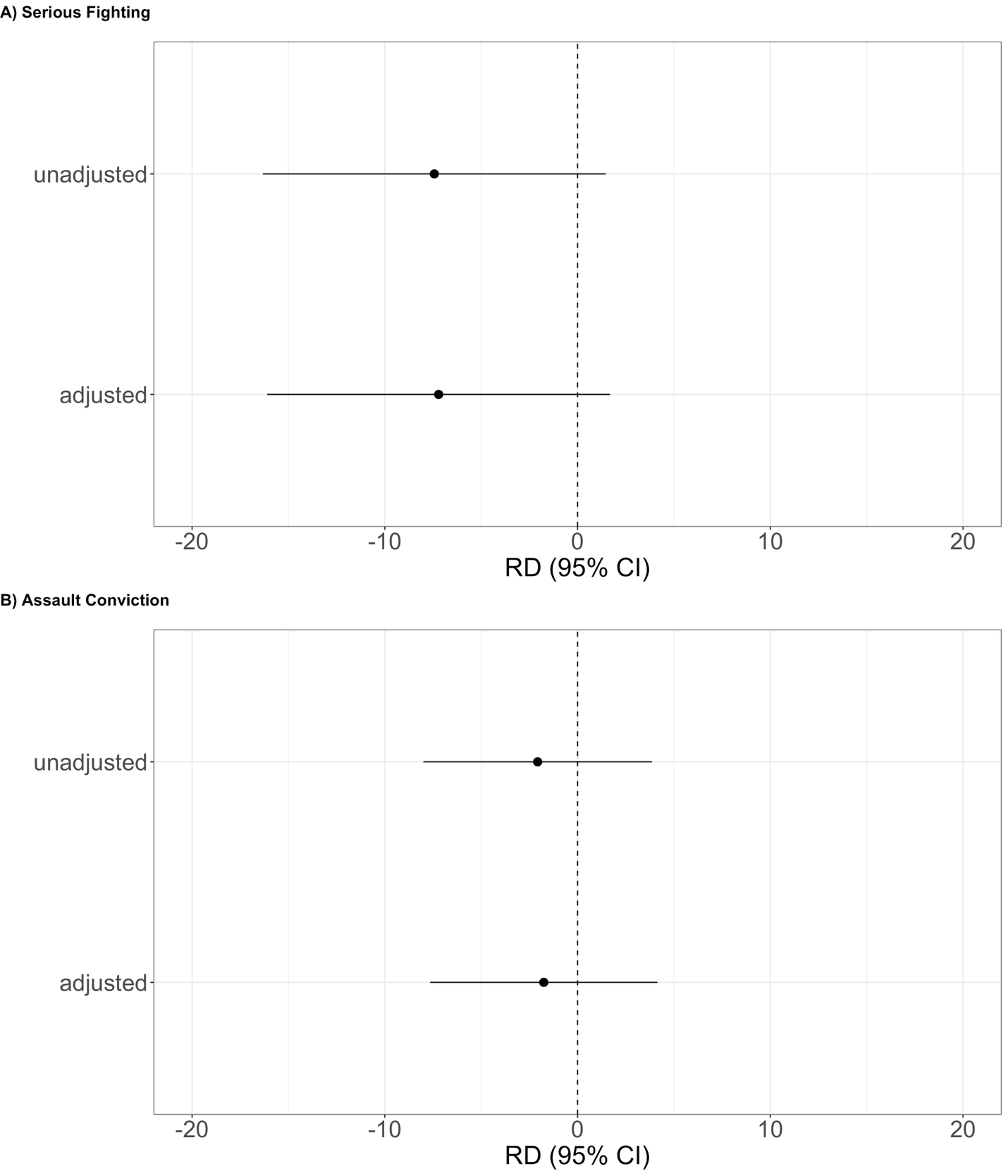


Adjusted estimates control for variables described in the text. All estimates control for heterogenous linear birth cohort trends for county Head Start adoption status.

### eFigure 6. Difference in Risk of Offspring Violence Associated with Maternal Head Start Exposure Among NLSCYA Respondents Whose Maternal Grandmother Had Less Than a High School Degree, By Intersectional Social Identities *(Additive Scale)*


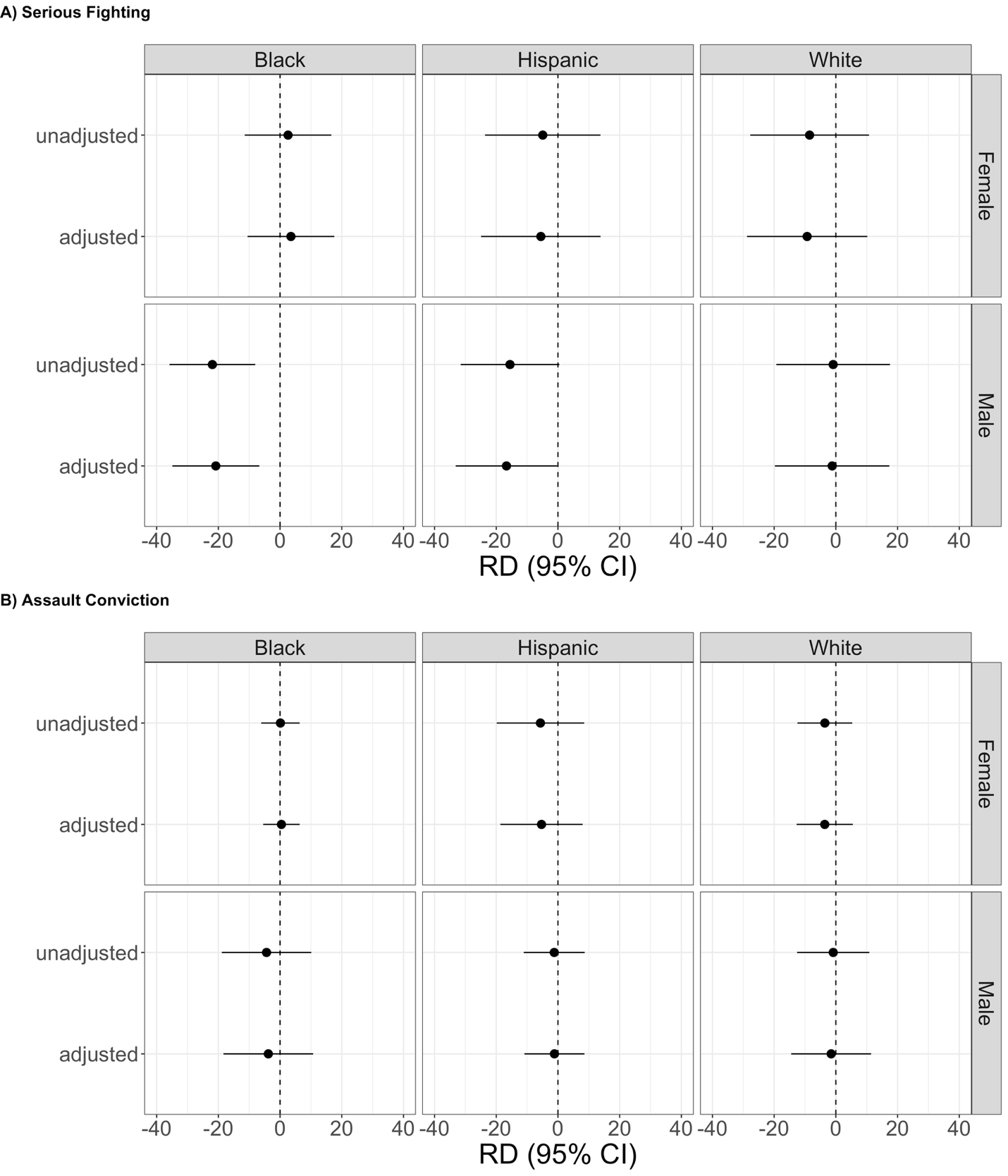


Estimates control for variables described in the text. All estimates control for heterogenous linear birth cohort trends for county Head Start adoption status.

### eFigure 7. Relative Risk of Offspring Serious Fighting Associated with Maternal Head Start Exposure Among NLSCYA Respondents Whose Maternal Grandmother Had a *High School Degree or Higher (Falsification Test)*


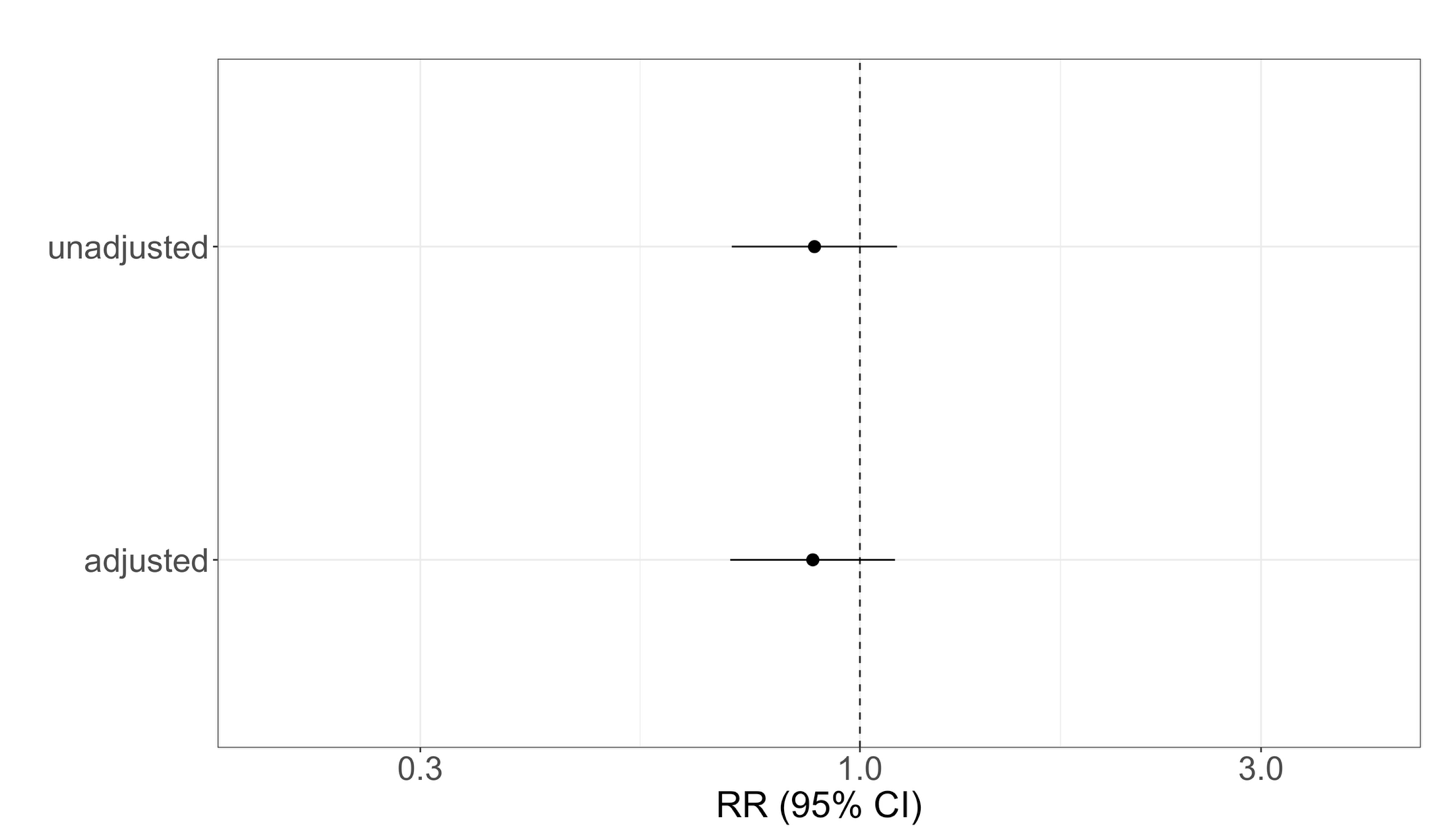
 Estimates for assault conviction were highly precise and are not shown. Adjusted estimates control for variables described in the text. All estimates control for heterogenous linear birth cohort trends for county Head Start adoption status.

### eFigure 8. Relative Risk of Offspring Serious Fighting Associated with Maternal Head Start Exposure Among NLSCYA Respondents Whose Maternal Grandmother Had a *High School Degree or Higher (Falsification Test)*, By Intersectional Social Identities


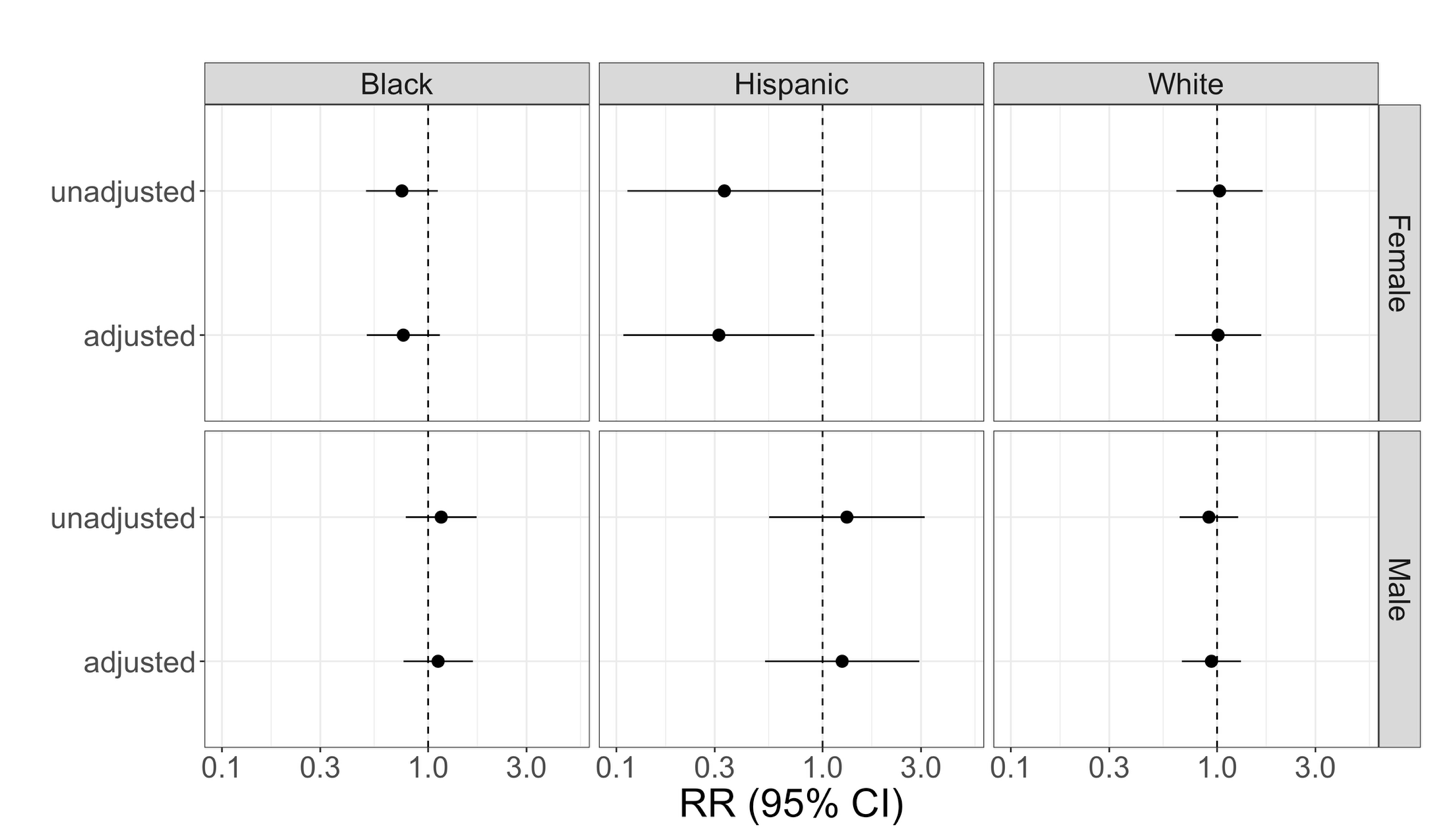


Estimates for assault conviction were highly precise and are not shown. Adjusted estimates control for variables described in the text. All estimates control for heterogenous linear birth cohort trends for county Head Start adoption status.

### References

1. US Census Bureau. What Updates to OMB’s Race/Ethnicity Standards Mean for the Census Bureau. Census.gov. April 8, 2024. Accessed April 11, 2025. https://www.census.gov/newsroom/blogs/random-samplings/2024/04/updates-race-ethnicity-standards.html

2. National Longitudinal Survey of Youth 1979. Race, Ethnicity & Immigration. Accessed July 19, 2025. https://www.nlsinfo.org/content/cohorts/nlsy79/topical-guide/household/race-ethnicity-immigration-data

3. US Bureau of Labor Statistics. Sex, National Longitudinal Surveys Children and Young Adults 1979. March 14, 2025. Accessed March 14, 2025. https://www.nlsinfo.org/content/cohorts/nlsy79-children/topical-guide/household/sex

4. Bailey MJ, Sun S, Timpe B. Prep School for Poor Kids: The Long-Run Impacts of Head Start on Human Capital and Economic Self-Sufficiency. *Am Econ Rev*. 2021;111(12):3963-4001. doi:10.1257/aer.20181801

5. Bailey M, Goodman-Bacon A. Replication data for: The War on Poverty’s Experiment in Public Medicine: Community Health Centers and the Mortality of Older Americans. Published online October 11, 2019. doi:10.3886/E112871V1

6. Barr A, Gibbs C. Breaking the Cycle? Intergenerational Effects of an Anti-Poverty Program in Early Childhood. *J Polit Econ*. Published online May 9, 2022. doi:10.1086/720764

7. Hernán M, Robins J. *Causal Inference: What If*. Chapman & Hall/CRC; 2020.

8. Petersen ML, Sinisi SE, van der Laan MJ. Estimation of Direct Causal Effects. *Epidemiology*. 2006;17(3):276. doi:10.1097/01.ede.0000208475.99429.2d

9. Stavola BLD, Herle M, Pickles A. Framing Causal Questions in Life Course Epidemiology. *Annu Rev Stat Its Appl*. 2022;9(Volume 9, 2022):223-248. doi:10.1146/annurev-statistics-040120-024748

10. Rubin DB. *Multiple Imputation for Nonresponse in Surveys*. Vol 81. John Wiley & Sons; 2004.
